# Systematic Review and Meta-Analysis of Intensive Care Unit Scoring Systems’ Performance in Patients with Pre-Existing Kidney Disease

**DOI:** 10.1016/j.ekir.2026.106560

**Published:** 2026-04-22

**Authors:** Hajar El-wadia, Marisha Karim, Amos Buh, Abdulghani O. Kabli, Nandini Biyani, Risa Shorr, Inok Lee, Deena Fremont, Ron Wald, Shane W. English, Samuel A. Silver, Ayub Akbari, Gregory A. Knoll, Edward G. Clark, Gregory L. Hundemer

**Affiliations:** 1 School of Epidemiology and Public Health, University of Ottawa, Ottawa, Ontario, Canada; 2 Inflammation and Chronic Disease Program, Ottawa Hospital Research Institute, Ottawa, Ontario, Canada; 3 Department of Critical Care Medicine, King Faisal Specialist Hospital and Research Center, Jeddah, Saudi Arabia; 4 Learning Services, The Ottawa Hospital, Ottawa, Ontario, Canada; 5 Department of Medicine, Division of Nephrology, University of Ottawa, Ottawa, Ontario, Canada; 6 Division of Nephrology, Department of Medicine, St. Michael’s Hospital, Toronto, Ontario, Canada; 7 Department of Nephrology and Hypertension, Tel Aviv Sourasky Medical Center, Tel Aviv, Israel; 8 Division of Critical Care, Department of Medicine, University of Ottawa, Ottawa, Ontario, Canada; 9 Acute Care Research, Ottawa Hospital Research Institute, Ottawa, Ontario, Canada; 10 Division of Nephrology, Department of Medicine, Queen’s University, Kingston, Ontario, Canada

**Keywords:** APACHE, CKD, ESKD, ICU, SAPS, SOFA

## Abstract

**Introduction:**

Several scoring systems are used to provide prognostic estimates of mortality among patients admitted to the intensive care unit (ICU) but do not account for baseline kidney function. This systematic review and meta-analysis evaluates the performance of commonly used ICU scoring systems in patients with nondialysis chronic kidney disease (CKD), end-stage kidney disease (ESKD) on maintenance dialysis, and kidney transplant recipients (KTRs).

**Methods:**

We searched Ovid Medline, Embase, and Scopus from inception through October 10, 2024, to identify observational studies reporting the prognostic performance of ICU scoring systems for mortality in patients with pre-existing kidney disease, analyzed independently or compared with a general ICU population.

**Results:**

Twelve studies involving 578,027 patients met inclusion criteria, evaluating 5 ICU scoring systems (sequential organ failure assessment [SOFA], acute physiologic and chronic health evaluation [APACHE] II/III, and simplified acute physiologic score [SAPS] II/III). Most studies involved patients with ESKD on maintenance dialysis and KTRs; data on patients with nondialysis CKD were very limited. For ESKD on maintenance dialysis, the area under the receiver operating characteristic curve (AUROC) showed excellent discrimination for all ICU scoring systems. However, discrimination was consistently lower in ESKD when compared with general ICU population, and calibration metrics showed consistent overestimation of mortality risk in ESKD. Among KTRs, scoring systems displayed consistently poor discrimination, for both SAPS III and SOFA, with variable calibration metrics.

**Conclusion:**

Common ICU scoring systems frequently overestimate mortality risk among patients with ESKD on dialysis and kidney transplantation. These findings highlight the need to develop or recalibrate scoring systems for patients with pre-existing kidney disease.

## Graphical abstract

**Figure:**
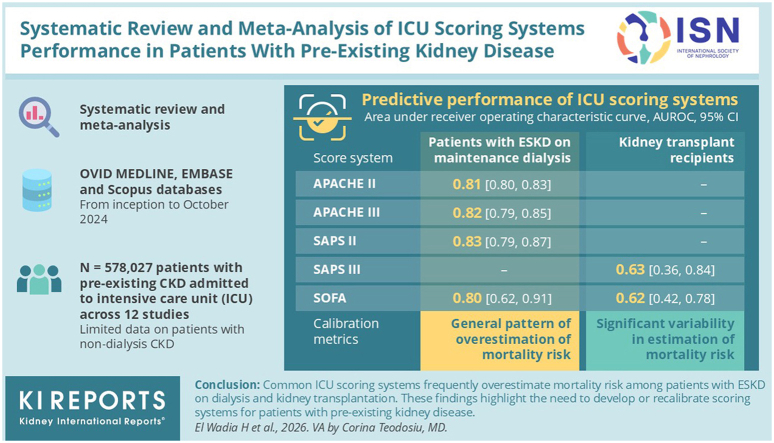

Several prognostic scoring systems have been developed to measure severity of illness and predict outcomes for patients admitted to the ICU. Commonly used and validated scoring systems include APACHE,^1^, ^2^, ^3^, ^4^, ^5^, ^6^, ^7^, ^8^ SAPS,^1^^,^^9^ and SOFA.^10^ These scoring systems use information including comorbidities, admission diagnosis, physiologic data, and laboratory measurements to provide a numerical severity of illness score that estimates mortality risk, as well as length of stay in some instances. In addition to estimating mortality risk, they play important roles in clinical trial design by ensuring comparable baseline risks between groups,^11^ healthcare system assessment of quality care benchmarks,^12^ and allocation of limited hospital resources.^13^

Although these commonly used prognostic ICU scoring systems were developed for general ICU populations, their performance may differ in specific patient populations including ethnic minorities^14^ and in different clinical scenarios including cardiac surgery,^15^, ^16^, ^17^ acute myocardial infarction,^18^ post-cardiac arrest,^19^ solid organ transplant,^20^ cancer,^21^ HIV,^22^ COVID-19,^23^ and pregnancy.^24^ As a result, specialized ICU scoring systems have been designed for some of these specialized patient populations.^25^^,^^26^ However, there is limited data on patients with pre-existing kidney disease, including nondialysis CKD, ESKD on maintenance dialysis, and kidney transplantation. Notably, patients with pre-existing kidney disease are at increased risk for ICU admission with patients of ESKD having an estimated 25- to 30-fold higher risk of ICU admission relative to the general ICU population.^27^ Most of these scoring systems incorporate some measure of kidney function, usually by assessing serum creatinine or urine output on arrival to the ICU. However, they generally do not consider baseline kidney function, or they use a simple dichotomous yes or no label indicating the presence of baseline CKD or ESKD. Therefore, these scoring systems do not fully account for whether reduced kidney function in the ICU is a manifestation of severity of acute illness (i.e., acute kidney injury [AKI]) or simply a reflection of poor baseline kidney function, which may limit their performance in populations with pre-existing kidney disease. This systematic review and meta-analysis aims to evaluate the performance of commonly used prognostic ICU scoring systems among individuals with pre-existing kidney disease following current critical appraisal guidelines.^28^

## Methods

### Protocol

This systematic review and meta-analysis was conducted in accordance with the Preferred Reporting Items for Systematic Review and Meta-Analyses guidelines^28^^,^^29^ (see Supplementary Table S1 for the checklist). The protocol was registered *a priori* on the International Prospective Register of Systematic Reviews (registration number CRD42024611547) and was previously published.^29^ Deviations from the protocol along with explanations are provided in Supplementary Table S2.

### Data Sources and Searches

We searched Ovid MEDLINE, EMBASE, and Scopus from database inception to October 10, 2024 using a search strategy developed with the assistance of an information specialist and medical librarian (RS). The search strategy incorporated a combination of medical subject headings and keywords related to the ICU scoring systems, such as “*APACHE,*” “*SAPS,*” “*SOFA,*” “*mortality prediction,*” “*intensive care unit,*” “*organ dysfunction,*” “*critical care,*” and more. A complete description of the search strategy is provided in Supplementary Table S3.

### Eligibility Criteria

This review assessed observational studies involving adult patients (≥ 18 years) with pre-existing kidney disease (nondialysis CKD, ESKD on maintenance dialysis, or kidney transplantation) admitted to the ICU. Eligible studies evaluated the prognostic performance of commonly used ICU severity of illness scoring systems in estimating mortality outcomes, including but not limited to APACHE, SAPS, and SOFA. Studies were required to report performance metrics of the prognostic performance of ICU scoring systems for mortality in patients with pre-existing kidney disease either analyzed independently or compared with patients in ICU without pre-existing kidney disease. Studies were excluded if they did not report data separately for patients in ICU with pre-existing kidney disease, if they focused exclusively on AKI with or without pre-existing CKD, if severity of illness scoring systems were applied outside of ICU settings, or if materials were grey literature or conference abstracts.

### Study Selection

All retrieved articles were imported to Covidence for duplicate removal and screening. Five reviewers (HE-w, MK, AOK, NB, and IL) independently screened all titles and abstracts in duplicate. Any disagreements at this stage resulted in the inclusion of the article to full-text screening. Full-text articles were then screened in duplicate, with discrepancies resolved through consensus or arbitration by a third reviewer (AB). The reasons for exclusion were recorded after full-text screening. Reviewers were not blinded to the authors or journals when screening articles.

### Data Extraction and Quality Assessment

A standardized data extraction form was developed in Microsoft Excel and piloted by reviewers. Data were independently extracted, in duplicate, from full-text studies by 5 reviewers (HE-w, MK, AOK, NB, and IL). The extracted data included the following: (i) first author, year, and country, (ii) study design and sample size, (iii) description of baseline kidney disease (CKD [by stage], ESKD, kidney transplant), (iv) list of scoring systems evaluated, (v) ICU and hospital mortality, and (vi) reported performance metrics.

The methodological quality of the included studies was assessed using a standard critical appraisal tool from the Joanna Briggs Institute.^30^ Risk of bias for each study was assessed using the prediction model risk of bias assessment tool, which includes 4 domains (participants, predictors, outcomes, and analysis) relevant to prognostic model studies.^31^^,^^32^ Quality scores were summarized and certainty of the evidence for each outcome was rated according to the grading of recommendations assessment, development and evaluation framework as recommended by the most current guidelines for prognostic models’ assessment.

### Data Synthesis and Statistical Analysis

Data were synthesized on the prognostic performance for mortality of ICU scoring systems among patients with pre-existing kidney disease. The primary metrics of interest were discrimination and calibration. Discrimination refers to the accuracy of a given prognostic model (i.e., the ability to distinguish between survivors and nonsurvivors). Commonly reported discrimination metrics include the AUROC, and c-statistics. Calibration describes how a prognostic model performs over a wide spectrum of estimated mortalities (i.e., the agreement between the numbers of expected and observed outcome events across all outcome probabilities). Therefore, calibration provides insight about whether a given model underestimates or overestimates risk for a given population. Commonly reported calibration metrics include calibration slope/intercept, standardized mortality ratio, and the Hosmer-Lemeshow goodness-of-fit test.

### Meta-Analysis

The meta-analysis of AUROC values was conducted using random effects model and refined by conducting the pooling of estimates on the logit-transformed AUROC scale to improve statistical stability.^32^, ^33^, ^34^, ^35^ Random effects models were estimated using restricted maximum likelihood as implemented in the *meta version 8.2-0* package in R (version 4.3.3, R Foundation, Vienna, Austria)) within RStudio (version 2025.05.1 + 513, Posit PBC, Boston, MA). The pooled estimates were subsequently back-transformed to the AUROC scale for interpretation. For each study, the standard errors (SE) were derived from the reported 95% confidence interval (CI) using a normal approximation and included in the random effects model. Statistical heterogeneity was assessed using the I^2^, with conventional threshold values ≥ 50% considered indicative of substantial heterogeneity,^36^ and by computing 95% prediction intervals to reflect the expected range of AUROC values in new settings.^32^ Meta-analysis of calibration was not feasible as studies varied in whether calibration was reported and in statistical methods used when reported (e.g., standardized mortality ratio, Hosmer-Lemeshow, calibration plots). The analysis code used in this study is publicly available on GitHub https://github.com/HajarEl24/R-Program/commit/fd6fa0fb870365e91aaf3b4a8f663e783edfc10d.

## Results

### Literature Search

**Figure 1:**
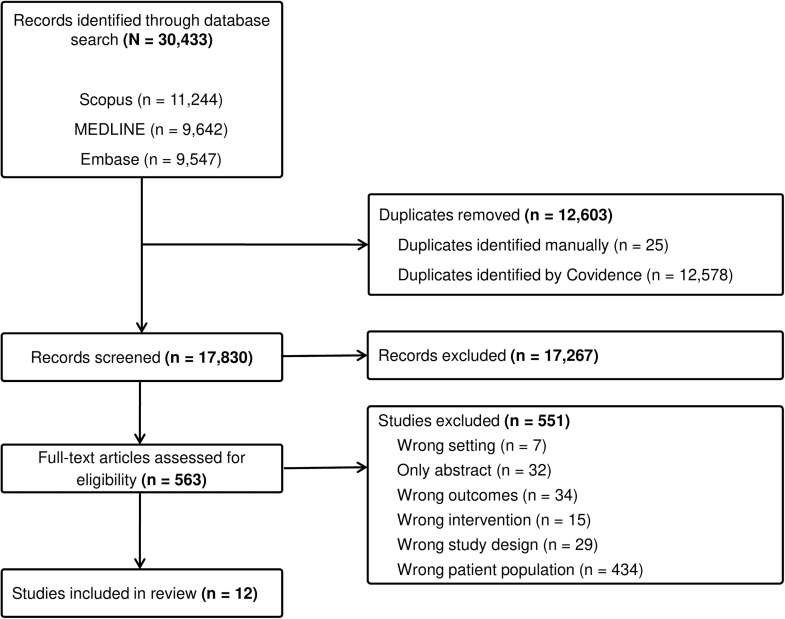
*PRISMA flow diagram detailing the study selection process.* PRISMA, Preferred Reporting Items for Systematic Review and Meta-Analyses.

A total of 30,433 results were identified using the databases search strategy (Figure 1). After the removal of duplicate results (*n* = 12,603), 17,830 unique citations underwent title and abstract screening. A total of 563 articles were then retrieved for full-text review. The most common reason for exclusion at this stage was ineligible patient population (*n* = 434), followed by wrong outcomes (*n* = 34), abstract-only publications (*n* = 32), and noneligible study designs (*n* = 29). A full listing of excluded studies and the reasons for exclusion are provided in Supplementary Table S4. Ultimately, 12 studies met all eligibility criteria and were included in this systematic review.

### Study Characteristics

Table 1^37^, ^38^, ^39^, ^40^, ^41^, ^42^, ^43^, ^44^, ^45^, ^46^ summarizes key characteristics of the included studies. A total of 578,027 patients admitted to ICU were assessed using the selected studies. This included 7368 patients with ESKD on maintenance dialysis and 1112 KTRs. Notably, there was very little information available on severity of illness score performance specifically among patients with nondialysis CKD with assessment in only 51 such patients. Individual study sample sizes ranged from 73 to 128,134 patients. The included studies were conducted worldwide across Australia (*n* = 1), Brazil (*n* = 2), China (*n* = 1), France (*n* = 1), India (*n* = 2), Japan (*n* = 1), Pakistan (*n* = 1), Turkey (*n* = 1), and the United States (*n* = 2). Study populations included ESKD patients on maintenance dialysis only (*n* = 3),^37^^,^^38^^,^^41^ a mix of nondialysis CKD and ESKD patients (*n* = 2),^46^ KTRs only (*n* = 2),^42^^,^^47^ a mix of all solid organ transplant recipients (*n* = 1),^20^ and general ICU populations (*n* = 4).^39^^,^^40^^,^^43^^,^^45^

**Table 1:** Study characteristics

Study (Publication Yr)	Country	Cohort study design	ICU study population	Sample size, N	Sub- group sample size (*n*)	% Male	Mean (SD)/Median (IQR) age	Study period	ICU mortality (%)	Hospital mortality (%)	Scoring system(s)	Primary outcome	Secondary outcomes
Rana *et al.*^37^	Pakistan	Prospective	ESKD	189	ESKD *n* = 189	51	61 (13)	Jan 2021–Dec 2022	12.6	NA	SAPS II	ICU mortality	Number of organ system failure, MV duration
Shimada *et al.*^45^	Japan	Retrospective	All	128,134	ESKD *n* = 6,631 bNon-ESKD *n* = 121	62	71 (60–78)	Apr 2018–Mar 2021	All = 4 ESKD = 7.2 Non-ESKD = 3.8	All = 8.9 ESKD =18 Non-ESKD = 8.4	APACHE II APACHE III SAPS II SOFA	Hospital mortality	ICU and hospital length of stay, discriminatory and calibration performance
Zhang *et al.*^42^	China	Retrospective	KTx recipients	428	KTx *n* = 428	59	55 (12)	NA	NA	NA	SOFA	90-day mortality	ICU length of stay, discriminatory performance of scoring system
Freitas *et al.*^47^	Brazil	Retrospective	KTx recipients	413	KTx *n* = 413	63	51 (14)	Sept 2013–June 2014	12.8	17.9	APACHE II SAPS III SOFA	Hospital mortality	ICU and hospital length of stay, ICU mortality, sepsis incidence, discriminatory and calibration performance of scoring systems
Goswami *et al.*^44^	India	Prospective	CKD and ESKD	100	ESKD *n* = 94 CKD (stage-4) *n* = 6	79	39 (14)^a^	Sept 2011–August 2013	34	39	APACHE II SAPS II SOFA	30-day mortality	ICU Length of stay, ICU mortality, discriminatory performance of scoring systems
Akbas *et al.* (2015)^46^	Turkey	Retrospective	All requiring CRRT	216	ESKD *n*= 101 AKI^a^*n* = 1s15 (2–5 CKD stages *n* = 45; NKF *n* = 70)	51	66 (53–73)	2000–2007	58	NA	APACHE II	ICU mortality	ICU length of stay, MV utilization, APACHE-II scores
Oliveira *et al.*^20^	Brazil	Prospective	Solid organ transplant recipients	501	KTx *n* = 271 Other solid organ transplant = 230	65.5	46 (2)	May 2006–Jan 2007	NA	2.6	APACHE II SAPS III	Hospital mortality	ICU and hospital length of stay, predicted mortality, and SMR
Juneja *et al.*^38^	India	Prospective	ESKD	73	ESKD *n* = 73	67	54 (15)	May 2007–July 2008	27	NA	APACHE II SOFA	30-day mortality	ICU mortality, ICU and hospital length of stay, predicted death rate, and SMR
Manhes *et al.*^39^	France	Prospective	All	1257	ESKD *n* = 92 Non-ESKD *n* = 1165	64	63 (15)	Jan 1996–Dec 1999	ESKD: 28 Non-ESKD: 22	ESKD = 38 Non-ESKD = 28	SAPS II	Hospital mortality	ICU mortality, ICU length of stay, 6-months mortality post ICU discharge, discriminatory and calibration performance of scoring systems
Dara *et al.*^40^	USA	Retrospective	All	32,612	ESKD *n* = 93 Non-ESKD *n* = 32,519	58	66 (54–76)	Jan 1999–Nov 2002	ESKD = 9 Non-ESKD = 5.5	ESKD = 16 Non-ESKD = 10	APACHE III SOFA	30-day mortality	ICU and hospital mortality, sepsis incidence, ICU length of stay
Uchino *et al.*^41^	Australia	Matched controls	All requiring CRRT	70	ESKD *n* = 38 Matched AKI, *n* = 32	60.5	ESKD = 45 (16) AKI = 55.5 (21)	3 mos	ESKD = 22 AKI = 38	ESKD = 34 AKI = 38	APACHE II SAPS II	ICU and Hospital mortality	ICU and hospital length of stay, MV duration
Clermont *et al.*^43^	USA	Prospective	All	1530	ESKD = 57 AKI = 254 Non-ESKD/No-AKI = 1219	NA	ESKD = 58 (2) AKI = 59 (1) Non-ESKD/ non-AKI =59 (1)	NA	ESKD = 11 AKI = 23 Non-ESKD/non-AKI = 5	ESKD = 14 AKI = 34 Non-ESKD/non-AKI = 9	APACHE III	ICU and hospital mortality	ICU length of stay, predicted mortality, and SMR

AKI, acute kidney injury; APACHE, Acute Physiology and Chronic Health Evaluation; CKD, non-dialysis chronic kidney disease; CRRT, continuous renal replacement therapy; ESKD, end-stage kidney disease on maintenance dialysis; ICU, intensive care unit; IQR, 25th–75th percentile interquartile range; KTx, kidney transplant recipient; MV, mechanical ventilation; NA, not available; SAPS, Simplified Acute Physiology Score; SMR, standardized mortality ratio; SOFA, Sequential Organ Failure Assessment.

a Study population may include some patients younger than 18 years.

b Non-ESKD = Individuals with or without CKD but not on maintenance dialysis and not having received a kidney transplant.

Across the 12 studies, 5 common ICU severity scoring systems were evaluated as follows: SOFA (*n* = 6), APACHE II (*n* = 7), SAPS II (*n* = 5), APACHE III (*n* = 3), and SAPS III (*n* = 2). All models incorporated kidney function indicators including creatinine, urine output, or blood urea nitrogen, although the timing and type of measurement varied. APACHE II, APACHE III, SAPS II, and SOFA scores are all calculated using the worst values recorded within the first 24 hours of ICU admission,^5^^,^^9^^,^^48^^,^^49^ whereas SAPS III is the only model using the worst value within the first hour of ICU admission for score calculation.^50^ Of these systems, only APACHE II incorporated chronic dialysis status as a dichotomous variable (Yes/No) under the chronic health component.^48^ However, none of the prognostic models assessed in this review included baseline creatinine or eGFR values. A detailed summary of each scoring system components is presented in Supplementary Table S5.

### Performance of ICU Scoring Systems in CKD and ESKD

Table 2 displays a summary of the nine studies focused on patients in ICU with ESKD on maintenance dialysis.^37^, ^38^, ^39^, ^40^, ^41^^,^^43^, ^44^, ^45^, ^46^ Five of the studies included both maintenance hemodialysis and peritoneal dialysis under the definition of ESKD, whereas 4 studies included only maintenance hemodialysis.^37^^,^^40^^,^^43^^,^^44^ Although Rana *et al.*^37^ and Clermont *et al.*^43^ did not report discrimination metrics, Rana *et al.*^37^ evaluated SAPS II in 189 patients with ESKD and found it was not a statistically significant factor in a multivariate analysis of mortality prognostication, whereas Clermont *et al.*^43^ reported calibration metrics only.^43^

**Table 2:** Performance metrics of prognostic scoring systems in patients with chronic kidney disease and end-stage kidney disease

Study	Subgroup sample size (*n*)	Scoring system	Mean (SD)/Median (IQR) Scores on ICU admission	Discrimination (AUROC [95% CI])	Calibration	Summary
Rana *et al.*^37^	ESKD *n*= 189	SAPS II	Survivors: 33.1 (11.3) Nonsurvivors: 69.3 (31.1)	NA	Not reported	SAPS II was included in the multivariate analysis and was not statistically significant.
Shimada *et al.*^45^	ESKD *n* = 6631 bNon-ESKD *n* = 121, 503	APACHE II	Overall = 14.0 (11–19) ESKD: 22.0 (18–26)	Non-ESKD = 0.89 (0.88–0.89) ESKD= 0.81 (0.80–0.83)	Calibration plot showed systematic overprediction in ESKD; acceptable fit in non-ESKD.	APACHE III showed better calibration than APACHE II. SOFA and SAPS II exhibited moderate overestimation of hospital mortality despite a good calibration slope. All four scoring systems discriminate mortality risk less effectively in patients with ESKD compared with non-ESKD.
		APACHE III	Overall = 53.0 (40–71) ESKD: 78.0 (67–92)	Non-ESKD= 0.90 (0.89–0.90) ESKD= 0.83 (0.82–0.85)	Slight overprediction in ESKD; good fit in non-ESKD.	
		SAPS II	Overall = 28.0 (20–40) ESKD: 43.0 (36–53)	Non-ESKD= 0.89 (0.89–0.90) ESKD= 0.81 [0.80–0.83]	Overprediction in ESKD; good fit in non-ESKD.	
		SOFA	Overall: 4.0 (2–7) ESKD: 8.0 (6–11)	Non-ESKD= 0.85 (0.84–0.85) ESKD= 0.79 (0.77–0.80)	Calibration plot shows close alignment between predicted and observed mortality in both ESKD and non-ESKD, with slight overprediction in ESKD	
Goswami *et al.*^44^	ESKD *n* = 94 CKD (stage-4) *n* = 6	APACHE II	28.2 (7.53)	0.96 (95% CI not reported)	NA	APACHE II, SAPS II, and SOFA showed excellent discriminatory power. However, no calibration metrics were reported, limiting further assessment of their predictive accuracy in patients with ESKD.
		SAPS II	43.0 (16.40)	0.99 (95% CI not reported)		
		SOFA	10.4 (5.20)	0.95 (95% CI not reported)		
Juneja *et al.*^38^	ESKD *n* = 73	APACHE II	NA	0.87 (0.78-0.95)	SMR = 0.48	APACHE II showed excellent discrimination but overpredicted 30-day mortality with SMR< 1. In contrast, SOFA displayed outstanding discrimination though no calibration assessment was reported for SOFA.
		SOFA	NA	0.92 (0.86–0.98)	NA	
Akbaş *et al.*^46^	ESKD *n*= 101 AKI^a^*n* = 115 (2-5 CKD stages *n* = 45; NKF *n* = 70)	APACHE II	ESKD = 27.5 (9) AKI = 28 (8)	ESKD = 0.78 (0.55–0.89) AKI = 0.52 (0.39–0.66)	NA	APACHE II showed acceptable discrimination in patients with ESKD compared with poor discrimination in AKI. Calibration was not assessed, and data were insufficient to infer mortality prediction accuracy.
Manhes *et al.*^39^	ESKD *n* = 92 Non-ESKD *n* = 1165	SAPS II	ESKD: 45 (21) Non-ESKD: 44 (25)	ESKD = 0.86 (0.82–0.90) Non-ESKD: NA	ESKD: Good calibration observed (38%) versus predicted mortality (41.6%). SMR = 0.91 Non-ESKD: NA	SAPS II demonstrated excellent discrimination, with calibration indicating no misfit. Mortality in patients with ESKD was slightly overestimated, as suggest by SMR < 1
Dara *et al.*^40^	ESKD *n* = 93 Non-ESKD *n* = 32,519	APACHE III	ESKD = 64.0 (47–79) Non-ESKD: NA	ESKD = 0.78 (0.68 – 0.86) Non-ESKD: NA	ESKD SMR = 0.74 Non-ESKD: NA	APACHE III demonstrated acceptable discrimination. SMR< 1 indicated overestimation of mortality in patients with ESKD. SOFA showed poor discrimination. Calibration was not reported.
		SOFA	ESKD = 6 (5–8)	ESKD = 0.66 (0.55 – 0.76)	NA	
Uchino *et al.*^41^	ESKD *n* = 38 Matched AKI, *n* = 32	APACHE II	ESKD = 22 (6) Matched AKI = 23 (9)	ESKD = 0.82 (0.65 – 0.92) Matched AKI: NA	SMR (ESKD) = 0.92 Matched AKI: NA	In patients with ESKD, both APACHE II and SAPS II showed an acceptable discriminatory power and slight overestimation of mortality SMR < 1.
		SAPS II	ESKD = 45 (13) Matched-AKI = 46 (17)	ESKD = 0.85 (0.68 – 0.95) Matched-AKI: NA	SMR (ESKD) = 0.92 Matched-AKI: NA	
Clermont *et al.*^43^	ESKD = 57 AKI = 254 Non-ESKD/No-AKI = 1219	APACHE III	ESKD = 64 (3) AKI = 64 (2) Non-ESKD/No-AKI = 42 (1)	NA	Calibration plot for ESKD patients displayed systematic overestimation of mortality across score ranges. SMR (ESKD) = 0.52	APACHE III discriminatory power not reported. Calibration plots showed consistent overestimation of mortality in patients with ESKD, with SMR < 1 confirming this trend.

AKI; acute kidney injury; AUROC, area under receiver operating characteristic curve; APACHE, Acute Physiology and Chronic Health Evaluation; CI, confidence interval; CKD, non-dialysis chronic kidney disease; ESKD, end-stage kidney disease on maintenance dialysis; ICU, intensive care unit; IQR, 25th–75th percentile interquartile range; NA, not available; NKF, normal kidney function; SAPS, Simplified Acute Physiology Score; SMR, standardized mortality ratios; SOFA, Sequential Organ Failure Assessment.

a This subgroup includes both acute-on-chronic kidney disease (not on dialysis at baseline) and normal kidney function as defined by normal baseline creatinine levels (≥ 90 ml/min per 1.73 m^2^).^46^^,^^51^

b Non-ESKD = Individuals with or without CKD but not on maintenance dialysis and not having received a kidney transplant.

#### Discrimination

Discriminatory performance was evaluated using AUROC (Table 2). Separated by ICU scoring system*,* AUROC values ranged as follows, from : (i) APACHE II = 0.78 (95% CI: 0.55–0.89)^46^ to 0.87 (95% CI: 0.78–0.95),^38^ (ii) APACHE III = 0.78 (95% CI: 0.68–0.86)^40^ to 0.83 (95% CI: 0.82–0.85),^45^ (iii) SAPS II = 0.81 (95% CI: 0.80–0.83)^45^ to 0.86 (0.82–0.90),^39^ and (iv) SOFA = 0.66 (95% CI: 0.66–0.76)^40^ to 0.92 (95% CI: 0.86–0.98),^38^ indicating acceptable to excellent discrimination.

In the largest comparative study, Shimada *et al.*^45^ observed that discrimination was consistently lower in patients with ESKD compared with patients in ICU having non-ESKD across multiple scoring systems (APACHE II, APACHE III, SAPS II, and SOFA). Full discrimination results for KTRs are presented in Table 3.

**Table 3:** Performance metrics of prognostic scoring systems in KTRs

Study	Subgroup Sample Size (n)	Scoring System	Median [IQR] Scores on ICU Admission	Discrimination (AUROC [95% CI])	Calibration	Summary
Zhang et al.^42^	KTx *n* = 428	SOFA Day 1	NA	0.52 (0.44–0.61)	NA	SOFA discrimination improved from Day 1 to Day 3 but remained overall poor, suggesting that later scores better predict 90-d mortality among ICU KTx patients.
		SOFA Day 2	NA	0.65 (0.56–0.75)		
		SOFA Day 3	NA	0.73 (0.63–0.83)		
Freitas et al.^47^	KTx *n* = 413	APACHE II	18 (14–23)	0.69 (0.62–0.76)	Calibration was poor with significant deviation between estimated and observed mortality. SMR = 0.65 (0.36 – 0.99)	Both APACHE II and SAPS III demonstrated poor discrimination and calibration. Calibration analysis displayed misfit and SMRs indicated APACHE II overestimated mortality risk, whereas SAPS III underestimated mortality. SOFA had acceptable discrimination, but calibration was not assessed
		SAPS III	47.5 (37–57)	0.73 (0.67–0.80)	Calibration was poor with significant deviation between estimated and observed mortality. SMR = 1.08 (0.6–1.65)	
		SOFA	5 (3–7)	0.71 (0.65–0.78)	Not reported	
Oliveira et al.^20^	KTx *n* = 271	APACHE II	NA	0.550^a^	SMR = 0.17 (0.08–0.31)	Both SAPS III and APACHE II demonstrated poor discrimination. SMRs suggest that APACHE II overestimated mortality, whereas SAPS III underestimated it. Calibration assessment using SMR indicates that APACHE II overestimated mortality risk, whilst SAPS III underestimated mortality.
		SAPS III	NA	0.46 (0.22–0.69)	SMR = 3.42 (1.37–7.04)	

AUROC, area under receiver operating characteristic curve; APACHE, Acute Physiology and Chronic Health Evaluation; CI, confidence interval; ICU, intensive care unit; IQR, 25th-75 percentile interquartile range; KTx, kidney transplant recipient; NA, not available; SAPS, Simplified Acute Physiology Score; SMR, standardized mortality ratio; SOFA, Sequential Organ Failure Assessment.

a The corresponding 95% CI reported in this study did not include this point estimate.

#### Calibration

Calibration metrics were reported in several studies (Table 2) and generally suggested overestimation of mortality risk (standardized mortality ratio < 1) in patients with ESKD. Separated by ICU scoring system, standardized mortality ratio values ranged, as follows, from: (i) APACHE II = 0.48^38^ – 0.92,^41^ (ii) APACHE III = 0.52^43^ – 0.74,^40^ and (iii) SAPS II = 0.91^39^ – 0.92.^41^ In addition, calibration slopes reported by Shimada *et al.*^45^ were lower in patients with ESKD than in patients with non-ESKD across multiple scoring systems, further demonstrating a pattern of mortality overestimation in this population. Additional calibration statistics, including Hosmer-Lemeshow results, are summarized in Table 2.

### Performance of ICU Scoring Systems in KTRs

Table 3 provides a summary of 3 studies assessing the prognostic performance of ICU severity of illness scoring systems among KTRs.

#### Discrimination

Across studies, discriminatory performance in KTRs was generally poor to modest, with AUROC values ranging from 0.46 (95% CI: 0.22–0.69)^20^ to 0.73 (95% CI: 0.67–0.80).^47^ One study by Zhang *et al.*^42^ reported some improvement in discrimination over time when using SOFA scores from day 1 to day 3 of ICU admission). However, overall discriminatory performance remained poor, and calibration metrics were not reported.^42^ Full discrimination results for KTRs are presented in Table 3.

#### Calibration

Calibration results were limited among the included studies and showed substantial variability in under and overestimation of mortality among KTRs. Full calibration results are provided in Table 3.

### Meta-Analysis

Overall, 9 of the included studies reported AUROC values for at least 1 severity of illness scoring system.^20^^,^^38^, ^39^, ^40^, ^41^, ^42^^,^^45^, ^46^, ^47^ However, 3 studies were excluded from the narrative analysis: 2 did not report AUROC metrics^37^^,^^43^ and another reported AUROC values without the corresponding 95% CI.^44^

**Figure 2:**
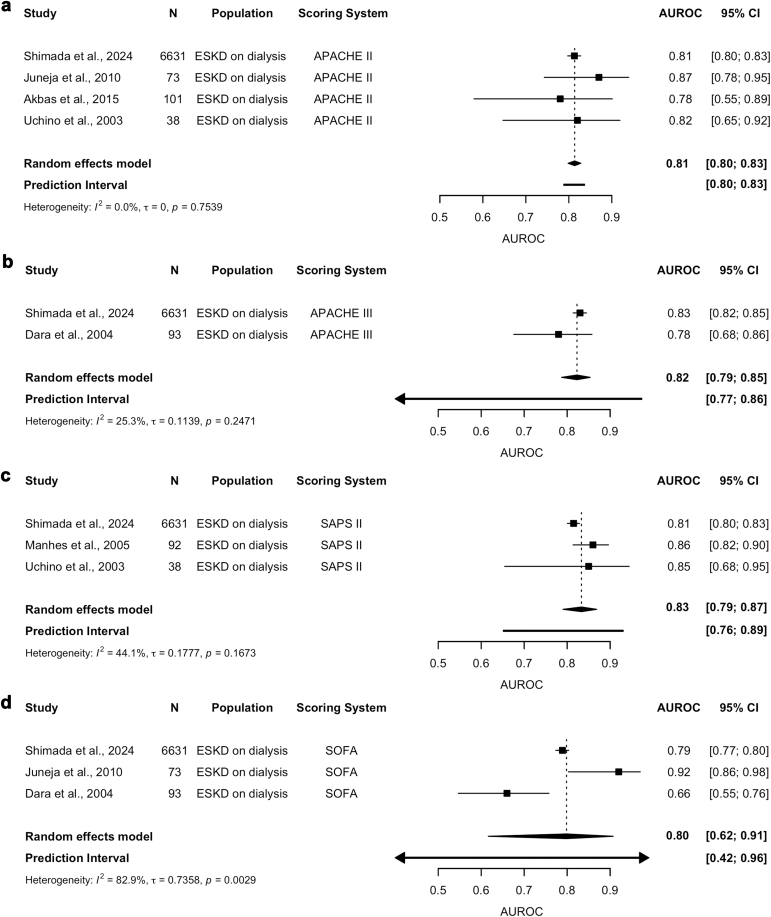
*AUROC estimates for discriminatory performance in patients with ESKD on maintenance dialysis admitted to the ICU.* Each forest plot has black boxes representing each study and its contribution to the overall estimate; horizontal lines represent the 95% CI; the diamonds represent the pooled estimates for each scoring systems as follows: (a) APACHE II, (b) APACHE III, (c) SAPS II, (d) SOFA. APACHE, Acute Physiology and Chronic Health Evaluation; AUROC, area under the receiver operating characteristic curve; CI, confidence interval; ESKD, end-stage kidney disease; ICU, intensive care unit; SAPS, Simplified Acute Physiology Score; SOFA, Sequential Organ Failure Assessment.

Among patients with ESKD on maintenance dialysis, the pooled estimates demonstrated that all evaluated scoring systems achieved excellent discriminatory accuracy (Figure 2a–d). The pooled AUROC for APACHE II, based on 4 studies, was 0.81 (95% CI: 0.80–0.83), with minimal between-study heterogeneity, reflected by low I^2^ and narrow prediction intervals (0.80–0.83).^38^^,^^45^^,^^46^^,^^52^ The pooled AUROC for APACHE III from 2 studies was 0.82 (95% CI: 0.79–0.85).^40^^,^^45^ SAPS II, pooled from 3 studies, had an AUROC of 0.83 (95% CI: 0.79–0.87).^39^^,^^45^^,^^52^ Both APACHE III and SAPS II had some heterogeneity as evidenced by the moderate I^2^ and wide prediction intervals (APACHE III [0.77–0.86] and SAPS II [0.76–0.89]). In contrast, the SOFA score with 3 studies demonstrated greater variability, with a pooled AUROC estimate of 0.80 (95% CI: 0.62–0.91) and significant heterogeneity (I^2^ = 82.9%), supported by wide prediction intervals (0.42–0.96).^38^^,^^40^^,^^45^ Certainty of evidence according to the grading of recommendations assessment, development and evaluation criteria for the pooled estimates was rated as low for APACHE II, APACHE III, and SAPS II, and very low for SOFA, largely because of the significant heterogeneity and inconsistency (Supplementary Table S6).

**Figure 3:**
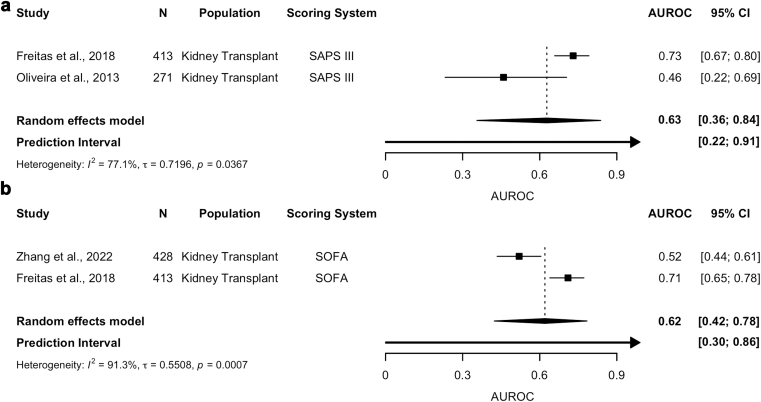
AUROC estimates for discriminatory performance in KTRs admitted to the ICU. Each forest plot has black boxes representing each study and its contribution to the overall estimate; horizontal lines represent the 95% CI; the diamonds represent the pooled estimates for each scoring systems as follows: (a) SAPS III, (b) SOFA. AUC, area under the curve; AUROC, area under the receiver operating characteristic curve; CI, confidence interval; ICU, intensive care unit; SAPS, Simplified Acute Physiology Score; SOFA, Sequential Organ Failure Assessment.

Among KTRs, the discriminatory performance was consistently poor (Figure 3a and b). The pooled AUROC for SAPS III across 2 studies was 0.63 (95% CI: 0.36–0.84; I^2^ = 77.1%), reflecting poor discriminatory accuracy with high heterogeneity.^20^, ^47^ Similarly, SOFA had poor discriminatory accuracy with a pooled AUROC of 0.62 (95% CI: 0.42–0.78; I^2^ = 91.3%) and substantial heterogeneity.^42^^,^^47^ Both models indicated significant variability across studies with wide prediction intervals (SAPS III [0.22–0.91], SOFA [0.30–0.86]). For KTRs, the certainty of evidence using the grading of recommendations assessment, development, and evaluation was rated very low for both models because of serious concerns with heterogeneity, inconsistency, and imprecision (Supplementary Table S6).

Meta-analysis was not performed among nondialysis patients of CKD because of a lack of studies assessing ICU prognostic scoring systems specifically among this population. Furthermore, meta-analysis of model calibration was not performed because studies varied substantially in whether calibration was assessed and, when it was, the metrics used were highly heterogeneous (see Tables 2 and 3 for how calibration metrics where reported, if any, by study).

### Risk of Bias

The methodological quality of the included studies, according to Joanna Briggs Institute appraisal scores, ranges from 62% to 90% suggesting moderate to high risk of bias (Supplementary Table S7). Complementing the Joanna Briggs Institute appraisal tool, PROBAST identified high risk of bias in most studies because of limitations in the analysis domain, specifically small sample sizes (< 100 outcome events), limited handling of missing data, and inconsistent reporting of calibration metrics (Supplementary Table S8). Supplementary Figure S1 provides a visual summary of these findings, highlighting overall high risk of bias in statistical analysis domain across the included studies.

## Discussion

This systematic review and meta-analysis identified 12 studies evaluating ICU scoring performance in patients with pre-existing kidney disease. These studies primarily focused on patients with ESKD on maintenance dialysis and KTRs with a few studies focused on the much larger nondialysis CKD population. Across studies, we found that ICU scoring systems (APACHE, SAPS, and SOFA) consistently overestimated mortality risk in patients with ESKD, despite maintaining adequate discriminatory performance in most cohorts.^38^^,^^39^^,^^41^^,^^44^, ^45^, ^46^ Among KTRs, these scoring systems displayed consistently poor discriminatory performance along with variable calibration metrics.^20^^,^^42^^,^^47^ These findings indicate that commonly used ICU scoring systems substantially misrepresent mortality risk prognosis among individuals with pre-existing kidney disease.

A central finding of this review is the lack of available data on the prognostic accuracy of ICU severity scores among patients with pre-existing kidney disease. This is highly relevant, as these patients comprise a substantial proportion of the ICU population. Previous studies have demonstrated that approximately 20% of patients admitted to the ICU have nondialysis CKD^53^ whereas 1% to 9% have ESKD.^54^ Despite this, we identified only 12 studies assessing the performance of these commonly used ICU scoring systems among patients with pre-existing kidney disease, many of which had relatively small sample sizes. Strikingly, there is a severe lack of data within the nondialysis CKD population which is, by far, the largest pre-existing kidney disease population in both the ICU and the population in general. We only identified 2 studies that specifically assessed the nondialysis CKD population – 1 study of 6 patients with stage 4 CKD which were pooled together with a larger population of ESKD patients and a second study of 45 patients^44^^,^^46^ with stages 2 to 5 CKD for which data on model performance was not provided. Therefore, a knowledge gap remains regarding the performance of these ICU scoring systems in the nondialysis CKD population and, more specifically, by CKD stage, which should be addressed by future research.

The tendency towards mortality risk overestimation in populations with pre-existing kidney disease may be best explained by the way kidney function is incorporated into these scoring systems. Most scores calculate risk using a single creatinine value or urine output measurements within the first 24 hours of ICU admission.^5^^,^^9^^,^^48^, ^49^, ^50^ Among patients with normal baseline kidney function, this may provide an accurate assessment of severity of illness which parallels the severity of AKI. However, for those with pre-existing kidney disease, this may not hold true. For instance, a patient with a baseline serum creatinine of 1.2 mg/dl whose ICU admission creatinine rises to 2.4 mg/dl would clearly meet criteria for stage 2 AKI. In contrast, a patient with a baseline creatinine of 2.1 mg/dl increasing to 2.4 mg/dl would have a similar admission value but represents a much smaller relative change in kidney function. Such severity scores may incorrectly presume the presence and/or severity of AKI leading to an overestimation of risk among CKD and ESKD populations. Although certain scoring systems such as APACHE II^48^ do incorporate a dichotomous yes or no variable about maintenance dialysis, this still fails to capture the wide spectrum of CKD. In KTRs, this problem is further compounded by the influence of immunosuppression (e.g., calcineurin inhibitors) and fluctuating graft function, which is not captured in traditional ICU scoring systems. Hence, the reliance on a single worst kidney function value on ICU admission rather than changes from baseline values to ICU admission predisposes to systematic inflation of mortality risk among these populations.

These findings have important implications. The consistent overestimation of mortality risk may contribute to an overly pessimistic approach to care of patients with pre-existing kidney disease, leading to premature limitation of therapy, the inappropriate denial of scarce life-saving therapies during a crisis, or misinforming families about prognosis. Moreover, benchmarking ICU outcomes using these scoring systems could introduce bias for hospitals that care for a higher proportion of CKD, ESKD, or kidney transplant patients, as the high mortality rates may reflect model miscalibration rather than true hospital performance.

It is important to remember that the ICU scoring systems evaluated in this study (APACHE, SAPS, SOFA) were derived from general ICU populations which represent a wide mix of patients based on demographics, social factors, and comorbidities. The original studies describing these scoring systems reported excellent discriminatory accuracy and acceptable calibration for their overall performance.^5^^,^^9^ Yet, subsequent evaluations of the original scoring systems demonstrated a deterioration in performance, especially calibration, when applied to new settings or special populations in the ICU.^55^^,^^56^ For instance, traditional scoring systems have shown reduced performance among cardiac surgery patients, spurring the development of cardiac-specific prognostic models, such as the cardiac surgery score,^57^ which show improved performance in this subgroup.

These findings beg the question of whether new or recalibrated ICU scoring systems may provide more accurate risk estimations for patients with pre-existing kidney disease. Such models could be designed for more reliable accounting of baseline kidney function in a standardized fashion (e.g., based on outpatient serum creatinine values when available) so changes in kidney function from baseline to ICU admission is captured. In turn, this may result in a number of downstream benefits including more accurate mortality risk estimation leading to more informed goals of care discussions, better resource allocation, and enhanced quality care benchmarking.

Our study is strengthened by a sensitive and comprehensive search strategy that covered several databases. We also acknowledge several limitations. First, several included studies were likely designed to address different research needs. Therefore, they did not comprehensively report on model performance metrics by specific pre-existing kidney disease populations, and many did not provide a nonkidney disease population as a comparator. Among the limited studies that did include both ESRD and non-ESRD groups, definitions of the non-ESRD populations varied substantially, limiting meaningful quantitative comparisons and requiring these findings to be presented at the individual study level. Second, performance metrics specifically for calibration were highly heterogeneous, leaving us unable to meta-analyze the calibration metrics. Third, there were only 2 studies which included a very limited number of nondialysis patients with CKD. In turn, we were not able to draw any substantial conclusions about how traditional ICU scoring systems perform in the broad nondialysis CKD population (or by CKD stage). This remains a major knowledge gap to be addressed in the future.

In summary, traditional ICU scoring systems show important limitations when applied to patients with pre-existing kidney disease. This includes a consistent overestimation of mortality risk among patients with ESKD on maintenance dialysis and overall poor discrimination and calibration among KTRs. Moreover, there is little to no data on how these systems perform in the nondialysis CKD population. Future work should include large multicenter external validation of ICU scoring systems across the complete spectrum of CKD and consideration for the development of novel or recalibrated scoring systems specifically designed for patients with pre-existing kidney disease.

## Disclosure

RW has received speaking fees and consulting fees from Vantive. SS has received speaking fees and an unrestricted educational grant from Vantive. All the other authors declared no competing interests.

## Supplementary Material

Supplementary file (PDF) **Figure S1.** Risk of Bias and applicability assessment of included studies using the PROBAST tool. **Table S1.** PRISMA 2020 checklist. **Table S2.** Deviations from protocol. **Table S3.** Search strategies. **Table S4.** List of excluded studies. **Table S5.** Characteristics of selected ICU severity of illness scoring systems and kidney-related parameters. **Table S6.** Statistical results summary of outcomes and GRADE assessment. **Table S7.** JBI critical appraisal results for cohort studies. **Table S8.** PROBAST risk of bias results.

## Footnotes

Supplementary file (PDF)
**Figure S1.** Risk of Bias and applicability assessment of included studies using the PROBAST tool. **Table S1.** PRISMA 2020 checklist. **Table S2.** Deviations from protocol. **Table S3.** Search strategies. **Table S4.** List of excluded studies. **Table S5.** Characteristics of selected ICU severity of illness scoring systems and kidney-related parameters. **Table S6.** Statistical results summary of outcomes and GRADE assessment. **Table S7.** JBI critical appraisal results for cohort studies. **Table S8.** PROBAST risk of bias results.

## References

[1] Capuzzo M., Valpondi V., Sgarbi A. (2000). Validation of severity scoring systems SAPS II and APACHE II in a single-center population. Intensive Care Med.

[2] Connors A.F., Dawson N.V., Desbiens N.A. (1995). A controlled trial to improve care for seriously III hospitalized patients: the study to understand prognoses and preferences for outcomes and risks of treatments (SUPPORT). JAMA.

[3] Escarce J.J., Kelley M.A. (1990). Admission source to the medical intensive care unit predicts hospital death independent of APACHE II score. JAMA.

[4] Ho K.M., Dobb G.J., Knuiman M., Finn J., Lee K.Y., Webb S.A.R. (2006). A comparison of admission and worst 24-hour Acute Physiology and Chronic Health Evaluation II scores in predicting hospital mortality: a retrospective cohort study. Crit Care Lond Engl.

[5] Knaus W.A., Wagner D.P., Draper E.A. (1991). The APACHE III prognostic system. Risk prediction of hospital mortality for critically ill hospitalized adults. Chest.

[6] Wagner D.P., Knaus W.A., Harrell F.E., Zimmerman J.E., Watts C. (1994). Daily prognostic estimates for critically ill adults in intensive care units: results from a prospective, multicenter, inception cohort analysis. Crit Care Med.

[7] Zimmerman J.E., Kramer A.A., McNair D.S., Malila F.M. (2006). Acute Physiology and Chronic Health Evaluation (APACHE) IV: Hospital mortality assessment for today’s critically ill patients. Crit Care Med.

[8] Zimmerman J.E., Kramer A.A., McNair D.S., Malila F.M., Shaffer V.L. (2006). Intensive care unit length of stay: benchmarking based on Acute Physiology and Chronic Health Evaluation (APACHE) IV. Crit Care Med.

[9] Le Gall J.R., Lemeshow S., Saulnier F. (1993). A new Simplified Acute Physiology Score (SAPS II) based on a European/North American multicenter study. JAMA.

[10] Vincent J.L., de Mendonça A., Cantraine F. (1998). Use of the SOFA score to assess the incidence of organ dysfunction/failure in intensive care units: results of a multicenter, prospective study. Working group on “sepsis-related problems” of the European Society of Intensive Care Medicine. Crit Care Med.

[11] Knaus W.A., Wagner D.P., Zimmerman J.E., Draper E.A. (1993). Variations in mortality and length of stay in intensive care units. Ann Intern Med.

[12] Afessa B., Keegan M.T., Hubmayr R.D. (2005). Evaluating the performance of an institution using an intensive care unit benchmark. Mayo Clin Proc.

[13] Zimmerman J.E., Wagner D.P., Knaus W.A., Williams J.F., Kolakowski D., Draper E.A. (1995). The use of risk predictions to identify candidates for intermediate care units. Implications for intensive care utilization and cost. Chest.

[14] Sarkar R., Martin C., Mattie H., Gichoya J.W., Stone D.J., Celi L.A. (2021). Performance of intensive care unit severity scoring systems across different ethnicities in the USA: a retrospective observational study. Lancet Digit Health.

[15] Barie P.S., Hydo L.J., Fischer E. (1995). Comparison of APACHE II and III scoring systems for mortality prediction in critical surgical illness. Arch Surg Chic Ill.

[16] Badreldin A.M.A., Doerr F., Ismail M.M. (2012). Comparison between Sequential Organ Failure Assessment score (SOFA) and Cardiac Surgery Score (CASUS) for mortality prediction after cardiac surgery. Thorac Cardiovasc Surg.

[17] Doerr F., Badreldin A.M.A., Can F., Bayer O., Wahlers T., Hekmat K. (2014). SAPS 3 is not superior to SAPS 2 in cardiac surgery patients. Scand Cardiovasc J SCJ.

[18] Muller G., Flecher E., Lebreton G. (2016). The ENCOURAGE mortality risk score and analysis of long-term outcomes after VA-ECMO for acute myocardial infarction with cardiogenic shock. Intensive Care Med.

[19] Salciccioli J.D., Cristia C., Chase M. (2012). Performance of SAPS II and SAPS III scores in post-cardiac arrest. Minerva Anestesiol.

[20] Oliveira VM de, Brauner J.S., Rodrigues Filho E. (2013). Is SAPS 3 better than APACHE II at predicting mortality in critically ill transplant patients?. Clin S Paulo Braz.

[21] Soares M., Salluh J.I.F. (2006). Validation of the SAPS 3 admission prognostic model in patients with cancer in need of intensive care. Intensive Care Med.

[22] Brown M.C., Crede W.B. (1995). Predictive ability of acute physiology and chronic health evaluation II scoring applied to human immunodeficiency virus-positive patients. Crit Care Med.

[23] Raschke R.A., Agarwal S., Rangan P., Heise C.W., Curry S.C. (2021). Discriminant accuracy of the SOFA score for determining the probable mortality of patients with COVID-19 pneumonia requiring mechanical ventilation. JAMA.

[24] Lewinsohn G., Herman A., Leonov Y., Klinowski E. (1994). Critically ill obstetrical patients: outcome and predictability. Crit Care Med.

[25] Tsaousi G.G., Pitsis A.A., Ioannidis G.D., Pourzitaki C.K., Yannacou-Peftoulidou M.N., Vasilakos D.G. (2015). Implementation of EuroSCORE II as an adjunct to APACHE II model and SOFA score, for refining the prognostic accuracy in cardiac surgical patients. J Cardiovasc Surg (Torino).

[26] Nassar Junior A.P., Mocelin A.O., Andrade F.M. (2013). SAPS 3, Apache IV or GRACE: which score to choose for acute coronary syndrome patients in intensive care units?. Sao Paulo Med J.

[27] Arulkumaran N., Annear N.M.P., Singer M. (2013). Patients with end-stage renal disease admitted to the intensive care unit: systematic review. Br J Anaesth.

[28] Page M.J., Moher D., Bossuyt P.M. (2021). PRISMA 2020 explanation and elaboration: updated guidance and exemplars for reporting systematic reviews. BMJ.

[29] Wadia H.E., Buh A., Kabli A.O. (2025). Effectiveness of predictive scoring systems in predicting mortality in relation to baseline kidney function in adult intensive care unit patients: a systematic review protocol. BMJ Open.

[30] Joanna Briggs Institute (JBI) (2020). https://jbi.global/critical-appraisal-tools.

[31] Moons K.G.M., Wolff R.F., Riley R.D. (2019). PROBAST: a tool to assess risk of bias and applicability of prediction model studies: explanation and elaboration. Ann Intern Med.

[32] Debray T.P.A., Damen J.A.A.G., Snell K.I.E. (2017). A guide to systematic review and meta-analysis of prediction model performance. BMJ.

[33] Damen J.A.A., Moons K.G.M., van Smeden M., Hooft L. (2023). How to conduct a systematic review and meta-analysis of prognostic model studies. Clin Microbiol Infect.

[34] Cochrane Chapter PDFs of the Cochrane Handbook for Systematic Reviews of Diagnostic Test Accuracy (v2.0). Cochrane. https://www.cochrane.org/authors/handbooks-and-manuals/handbook-systematic-reviews-diagnostic-test-accuracy/chapter-pdfs-cochrane-handbook-systematic-reviews-diagnostic-test-accuracy-v20.

[35] Cochrane Chapter 10: Analysing data and undertaking meta-analyses. https://www.cochrane.org/authors/handbooks-and-manuals/handbook/current/chapter-10#section-10-10-4.

[36] Higgins J.P.T., Thompson S.G. (2002). Quantifying heterogeneity in a meta-analysis. Stat Med.

[37] Rana M.A., Qayyum M.A., Khalid S.A. (2024). Clinical features and outcome of END-STAGE renal disease patients on maintenance hemodialysis admitted to medical intensive care unit. J Popul Ther Clin Pharmacol.

[38] Juneja D., Prabhu M.V., Gopal P.B., Mohan S., Sridhar G., Nayak K.S. (2010). Outcome of patients with end stage renal disease admitted to an intensive care unit in India. Ren Fail.

[39] Manhes G., Heng A.E., Aublet-Cuvelier B., Gazuy N., Deteix P., Souweine B. (2005). Clinical features and outcome of chronic dialysis patients admitted to an intensive care unit. Nephrol Dial Transplant.

[40] Dara S.I., Afessa B., Bajwa A.A., Albright R.C. (2004). Outcome of patients with end-stage renal disease admitted to the Intensive Care Unit. Mayo Clin Proc.

[41] Uchino S., Morimatsu H., Bellomo R., Silvester W., Cole L. (2003). End-stage renal failure patients requiring renal replacement therapy in the Intensive Care Unit: incidence, clinical features, and outcome. Blood Purif.

[42] Zhang H., Zhou Q., Ding Y. (2022). Sequential Organ Failure Assessment (SOFA) and 90-day mortality in patients with kidney transplant status at first ICU admission: a cohort study of 428 patients. Int Urol Nephrol.

[43] Clermont G., Acker C.G., Angus D.C., Sirio C.A., Pinsky M.R., Johnson J.P. (2002). Renal failure in the ICU: comparison of the impact of acute renal failure and end-stage renal disease on ICU outcomes. Kidney Int.

[44] Goswami J., Balwani M.R., Kute V., Gumber M., Patel M., Godhani U. (2018). Scoring systems and outcome of chronic kidney disease patients admitted in intensive care units. Saudi J Kidney Dis Transpl.

[45] Shimada H., Matsuoka Y., Miyakoshi C. (2024). Predictive performance of the sequential organ failure assessment score for in-hospital mortality in patients with end-stage kidney disease in intensive care units: a multicenter registry in Japan. Ther Apher Dial.

[46] Akbaş T., Karakurt S., Tuğlular S. (2015). Renal replacement therapy in the ICU: comparison of clinical features and outcomes of patients with acute kidney injury and dialysis-dependent end-stage renal disease. Clin Exp Nephrol.

[47] Freitas F.G.R., Lombardi F., Pacheco E.S. (2018). Clinical features of kidney transplant recipients admitted to the Intensive Care Unit. Prog Transplant.

[48] Knaus W.A., Draper E.A., Wagner D.P., Zimmerman J.E. (1985). Apache II: A severity of disease classification system. Crit Care Med.

[49] Vincent J.L., Moreno R., Takala J. (1996). The SOFA (Sepsis-related Organ Failure Assessment) score to describe organ dysfunction/failure. On behalf of the Working Group on Sepsis-Related Problems of the European Society of Intensive Care Medicine. Intensive Care Med.

[50] Metnitz P.G.H., Moreno R.P., Almeida E. (2005). SAPS 3--From evaluation of the patient to evaluation of the intensive care unit. Part 1: Objectives, methods and cohort description. Intensive Care Med.

[51] Levey A.S., Eckardt K.U., Tsukamoto Y. (2005). Definition and classification of chronic kidney disease: a position statement from Kidney Disease: improving Global Outcomes (KDIGO). Kidney Int.

[52] Uchino S., Kellum J.A., Bellomo R. (2005). Acute renal failure in critically ill patients: a multinational, multicenter study. JAMA.

[53] Lee Y., Kim T., Kim D.E. (2024). Differences in the incidence, characteristics, and outcomes of patients with acute kidney injury in the medical and surgical intensive care units. Kidney Res Clin Pract.

[54] Thompson S., Pannu N. (2012). Dialysis patients and critical illness. Am J Kidney Dis.

[55] Salluh J.I.F., Soares M. (2014). ICU severity of illness scores: APACHE, SAPS and MPM. Curr Opin Crit Care.

[56] Sawyer R.G., Durbin C.G., Rosenlof L.K., Pruett T.L. (1995). Comparison of Apache II scoring in liver and kidney transplant recipients versus trauma and general surgical patients in a single intensive-care unit. Clin Transpl.

[57] Becker R.B., Zimmerman J.E., Knaus W.A. (1995). The use of Apache III to evaluate ICU length of stay, resource use, and mortality after coronary artery by-pass surgery. J Cardiovasc Surg (Torino).

