# Supplementary material for: Systematic Review and Meta-Analysis of Intensive Care Unit Scoring Systems’ Performance in Patients with Pre-Existing Kidney Disease
Source: Kidney Int Rep. 2026 Apr 22;11(7):106560. doi: 10.1016/j.ekir.2026.106560 (PMC13226241; doi:10.1016/j.ekir.2026.106560)
Supplement: Supplementary file (PDF) — Figure S1. Risk of Bias and applicability assessment of included studies using the PROBAST tool. Table S1. PRISMA 2020 checklist. Table S2. Deviations from protocol. Table S3. Search strategies. Table S4. List of excluded studies. Table S5. Characteristics of selected ICU severity of illness scoring systems and kidney-related parameters. Table S6. Statistical results summary of outcomes and GRADE assessment. Table S7. JBI critical appraisal results for cohort studies. Table S8. PROBAST risk of bias results. [file mmc1.pdf]

SUPPLEMENTARY FILE: Performance of Commonly Used Scoring Systems in Predicting Mortality Among Adult Intensive Care Unit Patients with Pre-Existing Kidney Disease: A Systematic Review

Supplement Legend

Table S1. PRISMA 2020 checklist.

Table S2. Deviations from protocol.

Table S3. Search strategies.

Table S4. List of excluded studies.

Table S5. Characteristics of selected ICU severity of illness scoring systems and kidney-related parameters.

Table S6. Statistical results summary of outcomes and GRADE assessment.

Table S7. JBI critical appraisal results for cohort studies.

Table S8. PROBAST risk of bias results.

Figure S1. Risk of Bias and applicability assessment of included studies using the PROBAST tool.

Table S1. PRISMA 2020 checklist.

Section and Topic	Item #	Checklist item	Location where item is reported
**TITLE**			
Title	1	Identify the report as a systematic review.	Page 1
**ABSTRACT**			
Abstract	2	See the PRISMA 2020 for Abstracts checklist.	Line 46 – 72
**INTRODUCTION**			
Rationale	3	Describe the rationale for the review in the context of existing knowledge.	Background<br>Line 96 – 117
Objectives	4	Provide an explicit statement of the objective(s) or question(s) the review addresses.	Background<br>Line 114 – 117
**METHODS**			
Eligibility criteria	5	Specify the inclusion and exclusion criteria for the review and how studies were grouped for the syntheses.	Methods,<br>Eligibility Criteria<br>Line 137 – 149
Information sources	6	Specify all databases, registers, websites, organisations, reference lists and other sources searched or consulted to identify studies. Specify the date when each source was last searched or consulted.	Methods,<br>Data Sources and Searches<br>Line 128 – 135
Search strategy	7	Present the full search strategies for all databases, registers and websites, including any filters and limits used.	Supplementary File Table 3
Selection process	8	Specify the methods used to decide whether a study met the inclusion criteria of the review, including how many reviewers screened each record and each report retrieved, whether they worked independently, and if applicable, details of automation tools used in the process.	Methods<br>Study Selection<br>Line 151 – 158
Data collection process	9	Specify the methods used to collect data from reports, including how many reviewers collected data from each report, whether they worked independently, any processes for obtaining or confirming data from study investigators, and if applicable, details of automation tools used in the process.	Methods<br>Data Extraction and Quality Assessment<br>Line 160 – 166
Data items	10a	List and define all outcomes for which data were sought. Specify whether all results that were compatible with each outcome domain in each study were sought (e.g. for all measures, time points, analyses), and if not, the methods used to decide which results to collect.	Methods<br>Data Synthesis and Statistical Analysis<br>Line 177 – 189
	10b	List and define all other variables for which data were sought (e.g. participant and intervention	Methods

Section and Topic	Item #	Checklist item	Location where item is reported
		characteristics, funding sources). Describe any assumptions made about any missing or unclear information.	Data Synthesis and Statistical Analysis Line 177 – 189
Study risk of bias assessment	11	Specify the methods used to assess risk of bias in the included studies, including details of the tool(s) used, how many reviewers assessed each study and whether they worked independently, and if applicable, details of automation tools used in the process.	Methods<br>Data Extraction and Quality Assessment Line 168 – 175
Effect measures	12	Specify for each outcome the effect measure(s) (e.g. risk ratio, mean difference) used in the synthesis or presentation of results.	Methods<br>Data Synthesis and Statistical Analysis Line 177 – 189
Synthesis methods	13a	Describe the processes used to decide which studies were eligible for each synthesis (e.g. tabulating the study intervention characteristics and comparing against the planned groups for each synthesis (item #5)).	Methods<br>Data Synthesis and Statistical Analysis Line 177 – 189
	13b	Describe any methods required to prepare the data for presentation or synthesis, such as handling of missing summary statistics, or data conversions.	NA
	13c	Describe any methods used to tabulate or visually display results of individual studies and syntheses.	Methods<br>Data Extraction and Quality Assessment Line 165
	13d	Describe any methods used to synthesize results and provide a rationale for the choice(s). If meta-analysis was performed, describe the model(s), method(s) to identify the presence and extent of statistical heterogeneity, and software package(s) used.	Methods<br>Data Synthesis and Statistical Analysis Line 191 – 207
	13e	Describe any methods used to explore possible causes of heterogeneity among study results (e.g. subgroup analysis, meta-regression).	NA
	13f	Describe any sensitivity analyses conducted to assess robustness of the synthesized results.	NA
Reporting bias assessment	14	Describe any methods used to assess risk of bias due to missing results in a synthesis (arising from reporting biases).	NA
Certainty assessment	15	Describe any methods used to assess certainty (or confidence) in the body of evidence for an outcome.	Methods<br>Data Extraction and Quality Assessment<br>Line 168 – 175
**RESULTS**			
Study selection	16a	Describe the results of the search and selection process, from the number of records identified in the search to the number of studies included in the review, ideally using a flow diagram.	Results<br>Literature Search

Section and Topic	Item #	Checklist item	Location where item is reported
			Line 209 – 217<br>Figure 1. PRISMA flow diagram
	16b	Cite studies that might appear to meet the inclusion criteria, but which were excluded, and explain why they were excluded.	Supplementary Table 4. List of excluded studies
Study characteristics	17	Cite each included study and present its characteristics.	Results<br>Study Characteristics,<br>Line 219 – 244<br>Table 1. Study characteristics (pg. 35 – 36)
Risk of bias in studies	18	Present assessments of risk of bias for each included study.	Results,<br>Risk of Bias,<br>Line 357 - 365<br>Figure 2: AUROC estimates for discriminatory performance in patients with ESKD on maintenance dialysis admitted to the ICU,<br>Supplementary table 8: PROBAST Risk of bias results
Results of individual studies	19	For all outcomes, present, for each study: (a) summary statistics for each group (where appropriate) and (b) an effect estimate and its precision (e.g. confidence/credible interval), ideally using structured tables or plots.	Table 2: Performance Metrics of Predictive Scoring Systems in Patients with Chronic Kidney Disease and End-Stage Kidney Disease,<br>Table 3: Performance Metrics of Predictive Scoring Systems in Kidney Transplant

Section and Topic	Item #	Checklist item	Location where item is reported
			Recipients
Results of syntheses	20a	For each synthesis, briefly summarise the characteristics and risk of bias among contributing studies.	Results,<br>Performance of ICU Scoring Systems in Chronic Kidney Disease and End-Stage Kidney Disease,<br>Performance of ICU Scoring Systems in Kidney Transplant Recipients<br>Line 246 – 316
	20b	Present results of all statistical syntheses conducted. If meta-analysis was done, present for each the summary estimate and its precision (e.g. confidence/credible interval) and measures of statistical heterogeneity. If comparing groups, describe the direction of the effect.	Results,<br>Figures 2 & 3<br>Meta-analysis<br>Line 318 - 355
	20c	Present results of all investigations of possible causes of heterogeneity among study results.	NA
	20d	Present results of all sensitivity analyses conducted to assess the robustness of the synthesized results.	NA
Reporting biases	21	Present assessments of risk of bias due to missing results (arising from reporting biases) for each synthesis assessed.	NA
Certainty of evidence	22	Present assessments of certainty (or confidence) in the body of evidence for each outcome assessed.	Results,<br>Risk of bias<br>Line 357 – 365<br>Supplementary Figure 1
**DISCUSSION**			
Discussion	23a	Provide a general interpretation of the results in the context of other evidence.	Discussion<br>Line 379 – 441
	23b	Discuss any limitations of the evidence included in the review.	Discussion<br>Line 443 – 453
	23c	Discuss any limitations of the review processes used.	NA
	23d	Discuss implications of the results for practice, policy, and future research.	Discussion<br>Line 413 – 441
**OTHER INFORMATION**			

Section and Topic	Item #	Checklist item	Location where item is reported
Registration and protocol	24a	Provide registration information for the review, including register name and registration number, or state that the review was not registered.	Methods,<br>Protocol,<br>Line 124
	24b	Indicate where the review protocol can be accessed, or state that a protocol was not prepared.	Methods,<br>Protocol,<br>Line 122 - 125
	24c	Describe and explain any amendments to information provided at registration or in the protocol.	Supplementary table 2
Support	25	Describe sources of financial or non-financial support for the review, and the role of the funders or sponsors in the review.	Acknowledgements,<br>Funding,<br>Line 508 – 515
Competing interests	26	Declare any competing interests of review authors.	Acknowledgements,<br>Disclosures<br>Line 504 – 506
Availability of data, code and other materials	27	Report which of the following are publicly available and where they can be found: template data collection forms; data extracted from included studies; data used for all analyses; analytic code; any other materials used in the review.	Methods,<br>Data Synthesis and Statistical Analysis,<br>Line 205 – 207

From: Page MJ, McKenzie JE, Bossuyt PM, Boutron I, Hoffmann TC, Mulrow CD, et al. The PRISMA 2020 statement: an updated guideline for reporting systematic reviews. BMJ 2021;372:n71. doi: 10.1136/bmj.n71. This work is licensed under CC BY 4.0. To view a copy of this license, visit https://creativecommons.org/licenses/by/4.0/

Table S2. Deviations from protocol.

Deviation	Reason
1. Age eligibility, a small number of studies included cohorts with patients <18 years old that could not be separated. These studies were flagged for applicability in **Supplementary Table 5.**	Avoid discarding otherwise important metrics for ICU predictive scoring systems when age-stratified results were not available
2. PROBAST was used alongside JBI for risk of bias	PROBAST provides prediction model-specific risk of bias assessment
3. Meta-analysis for calibration metrics was not performed	High heterogeneity and variability
4. Subgroup analyses were not performed	Insufficient number of studies for each subpopulation

Table S3. Search strategies.

Ovid MEDLINE(R) ALL <1946 to October 10, 2024>

1	APACHE/ or Organ Dysfunction Scores/ or Simplified Acute Physiology Score/	9119
2	(organ adj2 (dysfunction or failure) adj2 (assessment* or score*)).tw,kf.	7719
3	(sofas or sofa score* or mods or lods).tw,kf.	13884
4	apache*.tw,kf.	11749
5	(Simplified Acute Physiology Score* or saps).tw,kf.	5089
6	(Mortality adj (scale* or score* or predict* or probab* or analysis)).tw,kf.	7915
7	Mortality Probability Model*.tw,kf.	139
8	"Acute Physiology And Chronic Health Evaluation".tw,kf.	6589
9	or/1-8	43118
10	Intensive Care Units/	75049
11	(intensive care or icu).tw,kf.	247225
12	critical* ill*.tw,kf.	75214
13	critical care/	62777
14	critical care.tw,kf.	47977
15	or/10-14	338244
16	9 and 15	19423
17	Mortality/	50396
18	(mortality or short term outcome* or long term outcome* or patient outcome* or predicted outcome*).tw,kf. or death*.ti.	1354113
19	17 or 18	1365788
20	16 and 19	13521
21	exp Cohort Studies/	2659722
22	(cohort* or retrospective* or prospective*).ti.	459786
23	(cohort* or retrospective* or prospective*).ab. /freq=2	694154
24	((cohort* or retrospective* or prospective*) adj3 (trial* or stud*)).tw,kf.	1270661
25	validation study/	112767
26	validat*.tw,kf.	846948
27	or/21-26	3947550
28	20 and 27	10248
29	(exp child/ or exp infant/) not exp adult/	1974124
30	((child* or infant* or pediatric or paediatric) not adult*).ti.	1285555
31	exp animals/ not humans/	5266257
32	(animal* or rat or rats or mouse or mice or pig or pigs or rabbit*).ti.	1845069
33	28 not (or/29-32)	9642

Scopus < Inception to 2024 October 10>

(((TITLE-ABS ("intensive care") OR TITLE-ABS (icu) OR TITLE-ABS ("critical* ill*") OR TITLE-ABS ("critical care")) AND ((TITLE-ABS (mortality W/1 TITLE-ABS (scale* OR score* OR predict* OR probab* OR analysis))) OR (TITLE-ABS ("Acute Physiology And Chronic Health Evaluation") OR TITLE-ABS ("mortality probability model*") OR TITLE-ABS ("Simplified Acute Physiology Score*") OR TITLE-ABS (saps) OR TITLE-ABS (sofas) OR TITLE-ABS ("sofa score") OR TITLE-ABS (mods) OR TITLE-ABS (lods) OR TITLE-ABS ("organ dysfunction score*") OR TITLE-ABS ("organ dysfunction assessment*") OR TITLE-ABS ("organ failure score*") OR TITLE-ABS ("organ failure assessment*") OR TITLE-ABS (apache)))) AND NOT (((TITLE (child* OR infant* OR pediatric OR paediatric) AND NOT TITLE (adult*)) OR ((animal* OR rat OR rats OR mouse OR mice OR pig OR pigs OR rabbit*) .ti.)))) AND (TITLE-ABS (prospective*) OR TITLE-ABS (retropective*) OR TITLE-ABS (prospective*) OR TITLE-ABS (cohort*) OR TITLE-ABS (validat*))

Embase Classic+Embase <1947 to 2024 October 10>

1	apache/ or simplified acute physiology score/	31094
2	organ dysfunction score/	1525
3	(organ adj2 (dysfunction or failure) adj2 (assessment* or score*)).tw.	10446
4	exp sequential organ failure assessment score/	19503
5	(sofas or sofa score* or mods or lods).tw.	21374
6	apache.tw.	22986
7	(Simplified Acute Physiology Score* or saps).tw.	8834
8	(Mortality adj (scale* or score* or predict* or probab* or analysis)).tw.	11968
9	Mortality Probability Model*.tw.	201
10	"Acute Physiology And Chronic Health Evaluation".tw.	8003
11	or/1-10	78349
12	intensive care unit/ or medical intensive care unit/ or neurological intensive care unit/ or surgical intensive care unit/	266761
13	(intensive care or icu).tw.	396061
14	critical* ill*.tw.	111039
15	intensive care/	156945
16	critical care.tw.	67149
17	or/12-16	608547
18	11 and 17	42838
19	*mortality/	127767
20	(mortality or short term outcome* or long term outcome* or patient outcome* or predicted outcome*).tw. or death*.ti.	2002433
21	19 or 20	2016076
22	18 and 21	28732
23	exp child/ not exp adult/	2653419
24	((child* or infant* or pediatric or paediatric) not adult*).ti.	1676056
25	(exp animal/ or nonhuman/ or animal experiment/) not exp human/	8343294
26	(animal* or rat or rats or mouse or mice or pig or pigs or rabbit*).ti.	2302423
27	or/23-26	11613905
28	22 not 27	27317
29	conference abstract.pt.	5253454
30	28 not 29	16630
31	*cohort analysis/	49850
32	*prospective study/	45082
33	*retrospective study/	40986
34	validation study/	115246
35	validat*.tw.	1193279
36	(cohort* or retrospective* or prospective*).ti. or (cohort* or retrospective* or prospective*).ab. /freq=2	1650886
37	((cohort* or retrospective* or prospective*) adj3 (trial* or stud*)).tw.	1926511
38	31 or 32 or 33 or 34 or 35 or 36 or 37	3626218
39	30 and 38	9547

Table S4. List of excluded studies.

Title	Author & Year	Reason for Exclusion
Comparison of Four Severity Assessment Scoring Systems in Critically Ill Patients for Predicting Patient Outcomes: A Prospective Observational Study From a Single Tertiary Center in Central India.	Mishra 2024	Wrong patient population;
APRICOT-Mamba: Acuity Prediction in Intensive Care Unit (ICU): Development and Validation of a Stability, Transitions, and Life-Sustaining Therapies Prediction Model.	Contreras 2024	Wrong outcomes;
The six scoring systems' prognostic value in predicting 24-hour mortality in septic patients.	Djikic 2024	Wrong patient population;
A novel prognostic model to predict mortality in patients with acute-on-chronic liver failure in intensive care unit.	Lin 2024	Wrong patient population;
Prognostic evaluation of quick sequential organ failure assessment score in ICU patients with sepsis across different income settings.	Li 2024	Wrong patient population;
Evaluating Prognostic Bias of Critical Illness Severity Scores Based on Age, Sex, and Primary Language in the United States: A Retrospective Multicenter Study.	Liu 2024	Wrong outcomes;
Modified Cardiovascular Sequential Organ Failure Assessment Score in Sepsis: External Validation in Intensive Care Unit Patients.	Ko 2023	Wrong patient population;
Validated Prognostic Scores to Predict Outcomes in ECLS-Bridged Patients to Lung Transplantation.	Faccioli 2023	Wrong patient population;
Evaluation of medication regimen complexity as a predictor for mortality.	Sikora 2023	Wrong intervention;
External validation of the ISARIC 4C Mortality Score to predict in-hospital mortality among patients with COVID-19 in a Canadian intensive care unit: a single-centre historical cohort study.	Vallipuram 2023	Wrong patient population;
Development and validation of a prediction model for in-hospital death in patients with heart failure and atrial fibrillation.	Yan 2023	Wrong patient population;
Prospective Evaluation of a Dynamic Acuity Score for Regularly Assessing a Critically Ill Patient's Risk of Mortality.	Kramer 2023	Wrong patient population;
Validation of the CLIF-C OF Score and CLIF-C ACLF Score to Predict Transplant-Free Survival in Patients with Liver Cirrhosis and Concomitant Need for Intensive Care Unit Treatment.	Nagel 2023	Wrong patient population;
Association of clinical prediction scores with hospital mortality in an adult medical and surgical intensive care unit in Kenya.	Brotherton 2023	Wrong patient population;
APACHE scoring as an indicator of mortality rate in ICU patients: a cohort study.	Mumtaz 2023	Wrong patient population;
A Comparison of ICU Mortality Scoring Systems Applied to COVID-19.	Monk 2023	Wrong patient population;
Risk factors for hospital mortality in intensive care unit survivors: a retrospective cohort study.	ESilva 2023	Wrong patient population;
Validation of the Acute Physiology and Chronic Health Evaluation (APACHE) II Score in COVID-19 Patients Admitted to the Intensive Care Unit in Times of Resource Scarcity.	Fernandes 2023	Wrong patient population;
Comparison of APACHE II and APACHE IV score as predictors of mortality in patients with septic shock in intensive care unit: A prospective observational study.	Bloria 2023	Wrong patient population;
Prognostic Performance of Sequential Organ Failure Assessment, Acute Physiology and Chronic Health Evaluation III, and Simplified Acute Physiology Score II Scores in Patients with Suspected Infection According to Intensive Care Unit Type.	Hwang 2023	Wrong patient population;
Validity of the total SOFA score in patients >= 80 years old acutely admitted to intensive care units: a post-hoc analysis of the VIP2 prospective, international cohort study.	Polok 2023	Wrong outcomes;
Investigating the Utility of the SOFA Score and Creating a Modified SOFA Score for Predicting Mortality in the Intensive Care Units in a Tertiary Hospital in Jordan.	Abu-Humaidan 2023	Wrong patient population;
Assessment of severity scoring systems for predicting mortality in critically ill patients receiving continuous renal replacement therapy.	Park 2023	Wrong patient population;
Automated APACHE II and SOFA score calculation using real-world electronic medical record data in a single center.	Mutchmore 2023	Wrong outcomes;
Modified National Early Warning Score (MNEWS) in predicting the mortality of intensive care unit patients.	Wang 2023	Wrong patient population;

A first-level customization study of SAPS II with Norwegian Intensive Care and Pandemic Registry (NIPaR) data.	Bruserud 2023	Wrong patient population;
External Validation of Mortality Prediction Models for Critical Illness Reveals Preserved Discrimination but Poor Calibration.	Cox 2023	Wrong patient population;
Variation of the SOFA score and mortality in patients with severe burns: A cohort study.	Calles 2023	Wrong patient population;
Comparison of Mortality Prediction Scores in Intermediate-Care Patients with Liver Cirrhosis at a German University Transplant Centre: A Prospective Study.	Jahn 2023	Wrong patient population;
Predictive value of the APACHE II score in cardiogenic shock patients treated with a percutaneous left ventricular assist device.	Mierke 2022	Wrong patient population;
Epidemiology and risk prediction of patients with severe burns admitted to a burn intensive care unit in a burn center in beijing: A 5-year retrospective study.	Wang 2022	Wrong patient population;
Comparison of mNUTRIC-S2 and mNUTRIC scores to assess nutritional risk and predict intensive care unit mortality.	Kim 2022	Wrong intervention;
Prediction of Inhospital Mortality in Critically Ill Patients With Sepsis: Confirmation of the Added Value of 24-Hour Lactate to Acute Physiology and Chronic Health Evaluation IV.	Baysan 2022	Wrong patient population;
Development and Internal Validation of a New Prognostic Model Powered to Predict 28-Day All-Cause Mortality in ICU COVID-19 Patients-The COVID-SOFA Score.	Moisa 2022	Wrong patient population;
Transplantation for EASL-CLIF and APASL acute-on-chronic liver failure (ACLF) patients: The TEA cohort to evaluate long-term post-Transplant outcomes.	Xia 2022	Wrong patient population;
Geriatric Nutritional Risk Index is Associated with Hospital Death in Elderly Patients with Multiple Organ Dysfunction Syndrome: A Retrospective Study Based on the MIMIC-III Database.	Mao 2022	Wrong patient population;
Comparison of Sequential Organ Failure Assessment Score and Sequential Organ Failure Assessment Score with pH in Outcome Prediction among ICU Patients: A Prospective Observational Study.	Agarwal 2022	Wrong outcomes;
Predictive Value of Sequential Organ Failure Assessment, Quick Sequential Organ Failure Assessment, Acute Physiology and Chronic Health Evaluation II, and New Early Warning Signs Scores Estimate Mortality of COVID-19 Patients Requiring Intensive Care Unit	Asmarawati 2022	Wrong patient population;
Data for validation and adjustment of APACHE II score in cardiogenic shock patients treated with a percutaneous left ventricular assist device.	Mierke 2022	Wrong patient population;
Modified Nutrition Risk in Critically Ill Score, A Prognostic Marker of Morbidity and Mortality in Mechanically Ventilated Patients: A Prospective Observational Study.	Dsouza 2022	Wrong intervention;
Cohort study of the APACHE II score and mortality for different types of intensive care unit patients.	Sungono 2022	Wrong patient population;
[Predictive value of sequential organ failure assessment on 28-day mortality in patients with post-cardiac arrest syndrome].	Lin 2022	Wrong patient population;
An improved prognostic model for predicting the mortality of critically ill patients: a retrospective cohort study.	Zhang 2022	Wrong patient population;
Accuracy of conventional disease severity scores in predicting COVID-19 ICU mortality: retrospective single-center study in Turkey.	Yildrim 2022	Wrong patient population;
Comparison of risk scoring systems for critical care patients with upper gastrointestinal bleeding: predicting mortality and length of stay.	Lincoln 2022	Wrong patient population;
Comparative Analysis of Composite Mortality Prediction Scores in Intensive Care Burn Patients.	Obed 2022	Wrong patient population;
Sequential organ failure assessment score as a predictor of the outcomes of patients hospitalized for classical or exertional heatstroke.	Yokoyama 2022	Wrong patient population;
Evaluation of mortality prediction using SOFA and APACHE IV tools in trauma and non-trauma patients admitted to the ICU.	KaramiNiaz 2022	Wrong patient population;
Relationships between RDW, NLR, CAR, and APACHE II scores in the context of predicting the prognosis and mortality in ICU patients.	Deniz 2022	Wrong patient population;
Predict models for prolonged ICU stay using APACHE II, APACHE III and SAPS II scores: A Japanese multicenter retrospective cohort study.	Takekawa 2022	Wrong outcomes;
Mortality prediction models for severe burn patients: Which one is the best?	Yazici 2022	Wrong patient population;

Internal Validation of the Predictive Performance of Models Based on Three ED and ICU Scoring Systems to Predict Inhospital Mortality for Intensive Care Patients Referred from the Emergency Department.	Rahmatinejad 2022	Wrong patient population;
Comparative Study of Sofa, Apache Ii, Saps Ii, as a Predictor of Mortality in Patients of Sepsis Admitted in Medical ICU.	Morkar 2022	Wrong patient population;
Association between the Predicted Value of APACHE IV Scores and Intensive Care Unit Mortality: A Secondary Analysis Based on EICU Dataset.	Xu 2022	Wrong patient population;
Sequential organ failure assessment score improves survival prediction for left ventricular assist device recipients in intensive care.	Chatterjee 2022	Only abstract ;
Association of Sequential Organ Failure Assessment (SOFA) components with mortality.	Polkki 2022	Wrong patient population;
Preintubation Sequential Organ Failure Assessment Score for Predicting COVID-19 Mortality: External Validation Using Electronic Health Record From 86 U.S. Healthcare Systems to Appraise Current Ventilator Triage Algorithms.	Keller 2022	Wrong patient population;
Predictive value of serial evaluation of the Sequential Organ Failure Assessment (SOFA) score for intensive care unit mortality in critically ill patients with COVID-19: a retrospective cohort study.	Gruyters 2022	Wrong patient population;
Superiority of Simplified Acute Physiologic Score II Compared with Acute Physiologic and Chronic Health Evaluation II and Sequential Organ Failure Assessment Scores for Predicting 48-Hour Mortality in Patients Receiving Continuous Kidney Replacement Thera	Jung 2022	Only abstract ;
Which scoring system is effective in predicting mortality in patients with Crimean Congo hemorrhagic fever? A validation study.	Bakir 2022	Wrong patient population;
Mortality prediction in intensive care units including premorbid functional status improved performance and internal validity.	Moser 2022	Wrong patient population;
SAPS III is superior to SOFA for predicting 28-day mortality in sepsis patients based on Sepsis 3.0 criteria.	Zhu 2022	Wrong patient population;
Multicenter International Cohort Validation of a Modified Sequential Organ Failure Assessment Score Using the Richmond Agitation-sedation Scale.	Rakhit 2022	Wrong patient population;
Retrospective Evaluation Of The Accuracy Of Five Different Severity Scores To Predict The Mortality In Burns Patients.	deCarvalho 2021	Wrong patient population;
Sequential organ failure assessment score is superior to other prognostic indices in acute pancreatitis.	Teng 2021	Wrong patient population;
Performance in mortality prediction of SAPS 3 And MPM-III scores among adult patients admitted to the ICU of a private tertiary referral hospital in Tanzania: a retrospective cohort study.	Kassam 2021	Wrong patient population;
ISARIC-4C Mortality Score overestimates risk of death due to COVID-19 in Australian ICU patients: a validation cohort study.	Durie 2021	Wrong patient population;
Validation of the Acute Physiology and Chronic Health Evaluation (APACHE) II and IV Score in COVID-19 Patients.	Vandenbrande 2021	Wrong patient population;
Utility of Acute Physiology and Chronic Health Evaluation (APACHE II) in Predicting Mortality in Patients with Pyogenic Liver Abscess: A Retrospective Study.	Lee 2021	Wrong patient population;
Development and validation of a nomogram to predict the mortality risk in elderly patients with ARF.	Xu 2021	Wrong intervention;
Characteristics and outcomes of patients admitted to adult intensive care units in Hong Kong: a population retrospective cohort study from 2008 to 2018.	Ling 2021	Wrong patient population;
Comparison of four prognostic scales for predicting mortality in patients with severe maternal morbidity.	JonguitudLopez 2021	Wrong patient population;
SAPS 3 in the modified NUTrition RIsk in the Critically ill score has comparable predictive accuracy to APACHE II as a severity marker.	Pasinato 2021	Wrong outcomes;
Comparison of mortality risk evaluation tools efficacy in critically ill COVID-19 patients.	Vicka 2021	Wrong patient population;
Comparison of General and Liver-Specific Prognostic Scores in Their Ability to Predict Mortality in Cirrhotic Patients Admitted to the Intensive Care Unit.	CostaESilva 2021	Wrong patient population;
Predictive capacity of prognostic scores for kidney injury, dialysis, and death in intensive care units.	Vasconcelos 2021	Wrong patient population;
Comparison of prognosis predictive value of 4 disease severity scoring systems in patients with acute respiratory failure in intensive care unit: A STROBE report.	Huang 2021	Wrong patient population;
Dynamic SOFA score assessments to predict outcomes after acute admission of octogenarians to the intensive care unit.	Loyrion 2021	Wrong patient population;

Intensive Care Unit Scoring Systems.	Pellathy 2021	Wrong patient population;
Performance of intensive care unit severity scoring systems across different ethnicities in the USA: a retrospective observational study.	Sarkar 2021	Wrong patient population;
Pilot analysis of the usefulness of mortality risk score systems at resuscitated patients.	Kiss 2021	Wrong patient population;
Evaluation and Validation of Four Scoring Systems: the APACHE IV, SAPS III, MPM0 II, and ICMM in Critically Ill Cancer Patients.	Siddiqui 2020	Wrong patient population;
Comparing Eight Prognostic Scores in Predicting Mortality of Patients with Acute-On-Chronic Liver Failure Who Were Admitted to an ICU: A Single-Center Experience.	Chen 2020	Wrong patient population;
External validation of a prognostic model for intensive care unit mortality: a retrospective study using the Ontario Critical Care Information System.	Priestap 2020	Wrong patient population;
Predicting hospital mortality for intensive care unit patients: Time-series analysis.	Awad 2020	Wrong patient population;
Validation of APACHE II, APACHE III and SAPS II scores in in-hospital and one year mortality prediction in a mixed intensive care unit in Poland: a cohort study.	Czajka 2020	Wrong patient population;
The applicability of commonly used predictive scoring systems in Indigenous Australians with sepsis: An observational study.	Hanson 2020	Wrong patient population;
Investigating SOFA, delta-SOFA and MPM-III for mortality prediction among critically ill patients at a private tertiary hospital ICU in Kenya: A retrospective cohort study.	Lukoko 2020	Wrong patient population;
Mortality Prediction Using SOFA Score in Critically Ill Surgical and Non-Surgical Patients: Which Parameter Is the Most Valuable?.	Fuchs 2020	Wrong patient population;
Derivation and validation of a new nutritional index for predicting 90 days mortality after ICU admission in a Korean population.	Son 2020	Wrong intervention;
The SAPS 3 score as a predictor of hospital mortality in a South African tertiary intensive care unit: A prospective cohort study.	vanderMerwe 2020	Wrong patient population;
Admission diagnosis and mortality risk prediction in a contemporary cardiac intensive care unit population.	Jentzer 2020	Wrong patient population;
Performance of three prognostic models in critically ill patients with cancer: a prospective study.	Martos-Benitez 2020	Wrong patient population;
Mortality prediction by SOFA score in ICU-patients after cardiac surgery; comparison with traditional prognostic-models.	Schoe 2020	Wrong patient population;
Comparison of a modified Sequential Organ Failure Assessment Score using RASS and FOUR.	Telles 2020	Wrong patient population;
Comparing the performance of SOFA, TPA combined with SOFA and APACHE-II for predicting ICU mortality in critically ill surgical patients: A secondary analysis.	Zhang 2020	Wrong patient population;
A new simplified and accurate sa-SOFA score.	Vacheron 2020	Wrong patient population;
The prognostic accuracy evaluation of SAPS 3, SOFA and APACHE II scores for mortality prediction in the surgical ICU: an external validation study and decision-making analysis.	Falcao 2019	Wrong patient population;
ACUTE PHYSIOLOGY AND CHRONIC HEALTH EVALUATION (APACHE) II SCORE - THE CLINICAL PREDICTOR IN NEUROSURGICAL INTENSIVE CARE UNIT.	Akavipat 2019	Wrong patient population;
External validation of the Simplified Mortality Score for the Intensive Care Unit (SMS-ICU).	Granholm 2019	Wrong patient population;
The use of APACHE II, SOFA, SAPS 3, C-reactive protein/albumin ratio, and lactate to predict mortality of surgical critically ill patients: A retrospective cohort study.	Basile-Filho 2019	Wrong patient population;
Acute Physiology and Chronic Health Evaluation II score for the assessment of mortality prediction in the intensive care unit: a single-centre study from Iran.	Bahtouee 2019	Wrong patient population;
Comparison of the accuracy of three early warning scores with SOFA score for predicting mortality in adult sepsis and septic shock patients admitted to intensive care unit.	Khwannimit 2019	Wrong patient population;
Modification of sequential organ failure assessment score using acute kidney injury classification.	Kotani 2019	Wrong study design;
Poison severity score and sequential organ failure assessment score: Carbon monoxide poisoning prognosis.	Wang 2019	Wrong outcomes;

Severity of illness assessment with application of the APACHE IV predicted mortality and outcome trends analysis in an academic cardiac intensive care unit.	Bennett 2019	Wrong patient population;
The Effectiveness of Scoring Systems in the Prediction of Diagnosis-Based Mortality.	Karagoz 2019	Wrong patient population;
Performance of SAPS II according to ICU length of stay: Protocol for an observational study.	Granholm 2019	Wrong patient population;
Survival prediction in intensive-care units based on aggregation of long-term disease history and acute physiology: a retrospective study of the Danish National Patient Registry and electronic patient records.	Nielsen 2019	Wrong patient population;
Predictors of mortality and validation of burn mortality prognostic scores in a Malaysian burns intensive care unit.	Lip 2019	Wrong patient population;
Characteristics of obstetric admissions to intensive care unit: APACHE II, SOFA and the Glasgow Coma Scale.	Fadiloglu 2019	Wrong patient population;
APACHE IV Score is Useful For Assessment and Stratification of Elderly Patients Over 65 Years With Acute Cholecystitis.	Jiang 2019	Wrong patient population;
Development of a new mortality scoring system for acute kidney injury with continuous renal replacement therapy.	Kim 2019	Wrong patient population;
The APACHE II Score as a Predictor of Mortality After Open Heart Surgery.	Yalcin 2019	Wrong patient population;
The association between the APACHE-II scores and age groups for predicting mortality in an intensive care unit: a retrospective study.	Edipoglu 2019	Wrong study design;
The predictive power of SAPS-3 and SOFA scores and their relations with patient outcomes in the Surgical Intensive Care Unit.	Mungan 2019	Wrong patient population;
Predictive Value of the Sequential Organ Failure Assessment Score for Mortality in a Contemporary Cardiac Intensive Care Unit Population.	Jentzer 2018	Wrong patient population;
[Performance evaluation of APACHE II and SAPS III in an intensive care unit].	Alvear-Vega 2018	Wrong patient population;
Validation of CLIF-C ACLF score to define a threshold for futility of intensive care support for patients with acute-on-chronic liver failure.	Engelmann 2018	Wrong patient population;
[APACHE II and SAPS II as predictors of brain death development in neurocritical care patients].	Rocchetti 2018	Wrong patient population;
[Evaluation value of the quick sequential organ failure assessment score on prognosis of intensive care unit adult patients with infection: a 17-year observation study from the real world].	Qin 2018	Wrong patient population;
Comparison of the predictive value of scoring systems on the prognosis of cirrhotic patients with suspected infection.	Lan 2018	Wrong patient population;
Intensive Care Decision Making: Using Prognostic Models for Resource Allocation.	Atashi 2018	Wrong outcomes;
Performance on the APACHE II, SAPS II, SOFA and the OHCA score of post-cardiac arrest patients treated with therapeutic hypothermia.	Choi 2018	Wrong patient population;
Does the clinical frailty score improve the accuracy of the SOFA score in predicting hospital mortality in elderly critically ill patients? A prospective observational study.	Langlais 2018	Wrong patient population;
Serial evaluation of SOFA and APACHE II scores to predict neurologic outcomes of out-of-hospital cardiac arrest survivors with targeted temperature management.	Yoon 2018	Wrong patient population;
Evaluation of ICU Risk Models Adapted for Use as Continuous Markers of Severity of Illness Throughout the ICU Stay.	Badawi 2018	Wrong study design;
Relevance of AND-ASPEN criteria of malnutrition to predict hospital mortality in critically ill patients: A prospective study.	Ceniccola 2018	Wrong patient population;
Development and internal validation of the Simplified Mortality Score for the Intensive Care Unit (SMS-ICU).	Granholm 2018	Wrong patient population;
qSOFA score: Predictive validity in Enterobacteriaceae bloodstream infections.	Burnham 2018	Wrong patient population;
Critical Illness Scoring Systems: Sequential Organ Failure Assessment, Acute Physiology and Chronic Health Evaluation II, and Quick Sequential Organ Failure Assessment to Predict the Clinical Outcomes in Scrub Typhus Patients with Organ Dysfunctions.	Balasubramanian 2018	Wrong patient population;
Mortality Prediction Using Acute Physiology and Chronic Health Evaluation II and Acute Physiology and Chronic Health Evaluation IV Scoring Systems: Is There a Difference?.	Venkataraman 2018	Wrong patient population;
Performance of APACHE IV in Medical Intensive Care Unit Patients: Comparisons with APACHE II, SAPS 3, and MPM0 III.	Ko 2018	Wrong patient population;

Single center validation of mortality scores in patients with acute decompensation of cirrhosis with and without acute-on-chronic liver failure.	Alexopoulou 2017	Wrong patient population;
Adaptation of the Acute Organ Failure Score for Use in a Medicare Population.	Courtright 2017	Wrong patient population;
Applicability of the APACHE II model to a lower middle income country.	Haniffa 2017	Wrong study design;
Performance of Simplified Acute Physiology Score 3 In Predicting Hospital Mortality In Emergency Intensive Care Unit.	Ma 2017	Wrong patient population;
Derivation and Validation of a Prognostic Model to Predict 6-Month Mortality in an Intensive Care Unit Population.	Hadique 2017	Wrong patient population;
Is 'gut feeling' by medical staff better than validated scores in estimation of mortality in a medical intensive care unit? - The prospective FEELING-ON-ICU study.	Radtke 2017	Wrong patient population;
Assessing contemporary intensive care unit outcome: development and validation of the Australian and New Zealand Risk of Death admission model.	Paul 2017	Wrong patient population;
Patients Admitted to Three Spanish Intensive Care Units for Poisoning: Type of Poisoning, Mortality, and Functioning of Prognostic Scores Commonly Used.	Banderas-Bravo 2017	Wrong patient population;
Validation of the Sepsis Severity Score Compared with Updated Severity Scores in Predicting Hospital Mortality in Sepsis Patients.	Khwannimit 2017	Wrong patient population;
Combining quick Sequential Organ Failure Assessment with plasma lactate concentration is comparable to standard Sequential Organ Failure Assessment score in predicting mortality of patients with and without suspected infection.	Ho 2017	Wrong patient population;
Effectiveness of the sequential organ failure assessment, acute physiology and chronic health evaluation II, and simplified acute physiology score II prognostic scoring systems in paraquat-poisoned patients in the intensive care unit.	Lee 2017	Wrong patient population;
Do Serially Recorded Prognostic Scores Predict Outcome Better Than One-Time Recorded Score on Admission? A Prospective Study in Adult Intensive Care Patients.	Manerikar 2017	Wrong study design;
A study on the efficacy of APACHE-IV for predicting mortality and length of stay in an intensive care unit in Iran.	Ghorbani 2017	Wrong patient population;
Comparison of acute physiology and chronic health evaluation II (APACHE II) and acute physiology and chronic health evaluation IV (APACHE IV) severity of illness scoring systems, in a multidisciplinary ICU.	Varghese 2017	Wrong patient population;
Comparison of six outcome prediction models in an adult burn population in a developing country.	Salehi 2017	Wrong patient population;
External validation of SAPS 3 and MPM0-III scores in 48,816 patients from 72 Brazilian ICUs.	Moralez 2017	Wrong patient population;
Comparison of APACHE II and SAPS II Scoring Systems in Prediction of Critically Ill Patients' Outcome.	Aminiahidashti 2017	Wrong patient population;
The Ability of the Acute Physiology and Chronic Health Evaluation (APACHE) IV Score to Predict Mortality in a Single Tertiary Hospital.	Choi 2017	Wrong patient population;
Predictive Performance of the Simplified Acute Physiology Score (SAPS) II and the Initial Sequential Organ Failure Assessment (SOFA) Score in Acutely Ill Intensive Care Patients: Post-Hoc Analyses of the SUP-ICU Inception Cohort Study.	Granholm 2016	Wrong patient population;
Mortality Prediction in Patients Admitted in Surgical Intensive Care Unit by Using APACHE IV.	Wetr 2016	Wrong patient population;
Simplified Acute Physiology Score II as Predictor of Mortality in Intensive Care Units: A Decision Curve Analysis.	Allyn 2016	Wrong patient population;
Comparing Time-Fixed Mortality Prediction Models and Their Effect on ICU Performance Metrics Using the Simplified Acute Physiology Score 3.	Engerstrom 2016	Wrong patient population;
Comparison of the accuracy and correctness of mortality estimates for Intensive Care Unit patients in internal clinics of the Czech Republic using APACHE II, APACHE IV, SAPS 3 and MPMoIII models.	Sedlon 2016	Wrong setting;
Predictive performance of quick Sepsis-related Organ Failure Assessment for mortality and ICU admission in patients with infection at the ED.	Wang 2016	Wrong patient population;
Various scoring systems for predicting mortality in Intensive Care Unit.	Evran 2016	Wrong patient population;
Predicting Mortality in Low-Income Country ICUs: The Rwanda Mortality Probability Model (R-MPM).	Riviello 2016	Wrong intervention;
Mortality prediction using TRISS methodology in the Spanish ICU Trauma Registry (RETRAUCI).	Chico-Fernandez 2016	Wrong intervention;

SOFA score to assess the severity of the post-cardiac arrest syndrome.	Cour 2016	Wrong patient population;
The predictive performance of the SAPS II and SAPS 3 scoring systems: A retrospective analysis.	Katsounas 2016	Only abstract ;
Validity of a Modified Sequential Organ Failure Assessment Score Using the Richmond Agitation-Sedation Scale.	Vasilevskis 2016	Wrong patient population;
Predictive scoring systems in multiorgan failure: A cohort study.	Sanchez-Casado 2016	Wrong patient population;
Comparison of mortality prediction models and validation of SAPS II in critically ill burns patients.	Pantet 2016	Wrong patient population;
Validation of a prognostic score for mortality in elderly patients admitted to Intensive Care Unit.	Sanchez-Hurtado 2016	Wrong patient population;
Comparison of Proposed Modified and Original Sequential Organ Failure Assessment Scores in Predicting ICU Mortality: A Prospective, Observational, Follow-Up Study.	GholipourBaradari 2016	Wrong patient population;
COMPARISON OF PATIENTS' ADMISSION, MEAN AND HIGHEST SOFA SCORES IN PREDICTION OF ICU MORTALITY: A PROSPECTIVE OBSERVATIONAL STUDY.	Baradari 2016	Wrong patient population;
Accuracy of SOFA score in prediction of 30-day outcome of critically ill patients.	Safari 2016	Wrong patient population;
Better prognostic marker in ICU - APACHE II, SOFA or SAP II!.	Naqvi 2016	Wrong patient population;
Sequential organ failure assessment scoring and prediction of patient's outcome in Intensive Care Unit of a tertiary care hospital.	Jain 2016	Wrong patient population;
Predicting mortality in the intensive care unit: a comparison of the University Health Consortium expected probability of mortality and the Mortality Prediction Model III.	Lipshutz 2016	Wrong patient population;
Evaluation of Probability of Survival using APACHE II & TRISS Method in Orthopaedic Polytrauma Patients in a Tertiary Care Centre.	Agarwal 2015	Wrong patient population;
Feasibility of transitioning from APACHE II to SAPS III as prognostic model in a Brazilian general intensive care unit. A retrospective study.	SerpaNeto 2015	Wrong patient population;
An External Independent Validation of APACHE IV in a Malaysian Intensive Care Unit.	Wong 2015	Wrong patient population;
Comparative evaluation of Acute Physiology and Chronic Health Evaluation II and Sequential Organ Failure Assessment scoring systems in patients admitted to the cardiac intensive care unit.	Argyriou 2015	Wrong setting;
Short-term outcomes and mortality after interhospital intensive care transportation: an observational prospective cohort study of 368 consecutive transports with a mobile intensive care unit.	Strauch 2015	Wrong setting;
Derivation and validation of the acute organ failure score to predict outcome in critically ill patients: a cohort study.	Elias 2015	Only abstract ;
Efficacy of the APACHE II score at ICU discharge in predicting post-ICU mortality and ICU readmission in critically ill surgical patients.	Lee 2015	Wrong patient population;
Comparing observed and predicted mortality among ICUs using different prognostic systems: why do performance assessments differ?.	Kramer 2015	Wrong patient population;
Use of APACHE II and SAPS II to predict mortality for hemorrhagic and ischemic stroke patients.	Moon 2015	Wrong patient population;
Comparison of mortality prediction models in burns ICU patients in Pinderfields Hospital over 3 years.	Douglas 2015	Wrong patient population;
Prognostic evaluation of patients undergoing living-donor liver transplant by APACHE II and MELD scores.	Zhang 2015	Wrong patient population;
Implementation of EuroSCORE II as an adjunct to APACHE II model and SOFA score, for refining the prognostic accuracy in cardiac surgical patients.	Tsaousi 2015	Wrong patient population;
Mortality risk prediction with an updated Acute Physiology and Chronic Health Evaluation II score in critically ill obstetric patients: a cohort study.	Paternina-Caicedo 2015	Wrong patient population;
Comparison of acute physiology and chronic health evaluation II and acute physiology and chronic health evaluation IV to predict intensive care unit mortality.	Parajuli 2015	Wrong study design;
Intensive care unit scoring systems outperform emergency department scoring systems for mortality prediction in critically ill patients: a prospective cohort study.	Moseson 2014	Wrong patient population;
Acute kidney injury enhances outcome prediction ability of sequential organ failure assessment score in critically ill patients.	Chang 2014	Wrong patient population;

Comparison of postinjury multiple-organ failure scoring systems: Denver versus Sequential Organ Failure Assessment.	Dewar 2014	Wrong patient population;
Scoring systems for 6-month mortality in critically ill cirrhotic patients: a prospective analysis of chronic liver failure - sequential organ failure assessment score (CLIF-SOFA).	Pan 2014	Wrong patient population;
The utility of scoring systems in critically ill cirrhotic patients admitted to a general intensive care unit.	Emerson 2014	Wrong patient population;
ICU severity of illness scores: APACHE, SAPS and MPM.	Salluh 2014	Wrong study design;
Glasgow Coma Scale score dominates the association between admission Sequential Organ Failure Assessment score and 30-day mortality in a mixed intensive care unit population.	Knox 2014	Only abstract ;
A calibration study of SAPS II with Norwegian intensive care registry data.	Haaland 2014	Wrong patient population;
Effectiveness of SAPS III to predict hospital mortality for post-cardiac arrest patients.	Bisbal 2014	Wrong patient population;
The Royal Free Hospital score: a calibrated prognostic model for patients with cirrhosis admitted to intensive care unit. Comparison with current models and CLIF-SOFA score.	Theocharidou 2014	Wrong patient population;
QT dispersion and prognostication of the outcome in acute cardiotoxicities: A comparison with SAPS II and APACHE II scoring systems.	Hassanian-Moghaddam 2014	Wrong outcomes;
Consideration of additional factors in Sequential Organ Failure Assessment score.	Lee 2014	Wrong outcomes;
Comparison of the Mortality Probability Admission Model III, National Quality Forum, and Acute Physiology and Chronic Health Evaluation IV hospital mortality models: implications for national benchmarking*.	Kramer 2014	Wrong study design;
External validation of the Simplified Acute Physiology Score (SAPS) 3 in Spain.	Lopez-Caler 2014	Only abstract ;
Predicting scores correlations in patients with septic shock - a cohort study.	Georgescu 2014	Wrong patient population;
Performance of the SAPS 3 admission score as a predictor of ICU mortality in a Philippine private tertiary medical center intensive care unit.	Hernandez 2014	Wrong patient population;
Validation of the APACHE IV model and its comparison with the APACHE II, SAPS 3, and Korean SAPS 3 models for the prediction of hospital mortality in a Korean surgical intensive care unit.	Lee 2014	Wrong patient population;
The ability of two scoring systems to predict in-hospital mortality of patients with moderate and severe traumatic brain injuries in a Moroccan intensive care unit.	Nejmi 2014	Wrong study design;
Assessment of performance and utility of mortality prediction models in a single Indian mixed tertiary intensive care unit.	Sathe 2014	Wrong patient population;
Utility of SOFA and APACHE II score in sepsis in rural set up MICU.	Desai 2013	Wrong patient population;
APACHE IV is superior to MELD scoring system in predicting prognosis in patients after orthotopic liver transplantation.	Hu 2013	Wrong patient population;
Risk prediction of hospital mortality for adult patients admitted to Australian and New Zealand intensive care units: development and validation of the Australian and New Zealand Risk of Death model.	Paul 2013	Wrong intervention;
Prediction of long-term mortality in ICU patients: model validation and assessing the effect of using in-hospital versus long-term mortality on benchmarking.	Brinkman 2013	Wrong patient population;
Prognostic scores for cirrhotic patients admitted to an intensive care unit: which consequences for liver transplantation?.	Galbois 2013	Wrong patient population;
Prospective comparison of three risk score models at three different surgical intensive care units.	Goertz 2013	Wrong patient population;
Clinical accuracy of RIFLE and Acute Kidney Injury Network (AKIN) criteria for predicting hospital mortality in critically ill patients with multi-organ dysfunction syndrome.	Ratanarat 2013	Wrong intervention;
Predictive value of outcome scores in patients suffering from cardiogenic shock complicating AMI: APACHE II, APACHE III, Elebute-Stoner, SOFA, and SAPS II.	Kellner 2013	Wrong patient population;
Why the surgical patients are so critical in their intensive care unit arrival?.	Basile-Filho 2013	Wrong patient population;
External validation of the Acute Physiology and Chronic Health Evaluation II in Korean intensive care units.	Kim 2013	Wrong patient population;
Assessment of mortality prediction models in a Ghanaian burn population.	Brusselaers 2013	Wrong patient population;

Assessing and combining repeated prognosis of physicians and temporal models in the intensive care.	Minne 2013	Wrong patient population;
Does the RIFLE Classification Improve Prognostic Value of the APACHE II Score in Critically Ill Patients?.	Wahrhaftig 2013	Wrong patient population;
A comparison of the Acute Physiology and Chronic Health Evaluation (APACHE) II score and the Trauma-Injury Severity Score (TRISS) for outcome assessment in Srinagarind Intensive Care Unit trauma patients.	Thanapaisal 2012	Wrong patient population;
Performance of APACHE III over time in Australia and New Zealand: a retrospective cohort study.	Paul 2012	Wrong patient population;
Prognostic performance of the Simplified Acute Physiology Score II in major Croatian hospitals: a prospective multicenter study.	Desa 2012	Wrong patient population;
Outcome prediction using clinical scores and biomarkers in patients with presumed severe infection in the emergency department.	Wilhelm 2012	Wrong patient population;
Predictors of mortality in patients successfully weaned from extracorporeal membrane oxygenation.	Chang 2012	Wrong patient population;
Performance assessment of Acute Physiology and Chronic Health Evaluation II and Simplified Acute Physiology Score II in a referral respiratory intensive care unit in Iran.	Fadaizadeh 2012	Wrong patient population;
Cross-validation of a Sequential Organ Failure Assessment score-based model to predict mortality in patients with cancer admitted to the intensive care unit.	Cardenas-Turanzas 2012	Wrong patient population;
Relationship between mortality and first-day events index from routinely gathered physiological variables in ICU patients.	Rivera-Fernandez 2012	Wrong intervention;
Prognostic models based on administrative data alone inadequately predict the survival outcomes for critically ill patients at 180 days post-hospital discharge.	Bohensky 2012	Wrong patient population;
Comparison between SAPS II and SAPS 3 in predicting hospital mortality in a cohort of 103 Italian ICUs. Is new always better?.	Poole 2012	Wrong patient population;
[NEMS: a new predictor of mortality in the critical patient?].	SimonGarcia 2012	Wrong patient population;
The utility of acute physiology and chronic health evaluation II scores for prediction of mortality among intensive care unit (ICU) and non-ICU patients with methicillin-resistant Staphylococcus aureus bacteremia.	Stevens 2012	Wrong patient population;
The outcome of critical illness in decompensated alcoholic liver cirrhosis.	Kavli 2012	Wrong patient population;
Sequential organ failure assessment score for evaluating organ failure and outcome of severe maternal morbidity in obstetric intensive care.	Oliveira-Neto 2012	Wrong patient population;
Severity scoring in the critically ill: part 1--interpretation and accuracy of outcome prediction scoring systems.	Breslow 2012	Wrong patient population;
Comparison of newer scoring systems with the conventional scoring systems in general intensive care population.	Juneja 2012	Wrong patient population;
A multicenter mortality prediction model for patients receiving prolonged mechanical ventilation.	Carson 2012	Wrong patient population;
Caution when using prognostic models: a prospective comparison of 3 recent prognostic models.	Nassar 2012	Wrong patient population;
A comparison of the performance of a model based on administrative data and a model based on clinical data: effect of severity of illness on standardized mortality ratios of intensive care units.	Brinkman 2012	Wrong patient population;
Clinical profile and predictors of mortality in patients of acute-on-chronic liver failure.	Garg 2012	Wrong patient population;
Comparison of the Sequential Organ Failure Assessment, Acute Physiology and Chronic Health Evaluation II scoring system, and Trauma and Injury Severity Score method for predicting the outcomes of intensive care unit trauma patients.	Hwang 2012	Wrong patient population;
Is the SAPS II score valid in surgical intensive care unit patients?.	Sakr 2012	Wrong patient population;
Mortality assessment in patients with severe acute pancreatitis: a comparative study of specific and general severity indices.	Amalio 2012	Wrong patient population;
Applicability of different scoring systems in outcome prediction of patients with mixed drug poisoning-induced coma.	EizadiMood 2011	Wrong patient population;
Role of APACHE score in predicting mortality in chest ICU.	Haidri 2011	Wrong patient population;
[The usefulness of severity scoring systems in elderly intensive care unit (ICU) patients with non-traumatic haemorrhagic shock].	Zajac 2011	Wrong patient population;

Acute physiology and chronic health evaluation II score is a better predictor of mortality than IBMP-10 in patients with ventilator-associated pneumonia.	Wiskirchen 2011	Wrong patient population;
Outcome scoring systems for short-term prognosis in critically ill cirrhotic patients.	Tu 2011	Wrong patient population;
Prognostic scores in cirrhotic patients admitted to a gastroenterology intensive care unit.	Freire 2011	Wrong patient population;
Performance of the third-generation models of severity scoring systems (APACHE IV, SAPS 3 and MPM-III) in acute kidney injury critically ill patients.	CostaeSilva 2011	Wrong patient population;
A comparison of the performance of Simplified Acute Physiology Score 3 with old standard severity scores and customized scores in a mixed medical-coronary care unit.	Khwannimit 2011	Wrong patient population;
A comparative study of four intensive care outcome prediction models in cardiac surgery patients.	Doerr 2011	Wrong patient population;
APACHE-II score correlation with mortality and length of stay in an intensive care unit.	Naved 2011	Wrong patient population;
Validation of the Simplified Acute Physiology Score 3 scoring system in a Korean intensive care unit.	Lim 2011	Wrong patient population;
External validation of Acute Physiology and Chronic Health Evaluation IV in Dutch intensive care units and comparison with Acute Physiology and Chronic Health Evaluation II and Simplified Acute Physiology Score II.	Brinkman 2011	Wrong patient population;
Severity of illness scoring systems in the intensive care unit.	Keegan 2011	Wrong study design;
A modified sequential organ failure assessment score for critical care triage.	Grissom 2010	Wrong patient population;
Prognostic scores in a gastroenterology intensive care unit.	Freire 2010	Wrong patient population;
Patients supported by extracorporeal membrane oxygenation and acute dialysis: acute physiology and chronic health evaluation score in predicting hospital mortality.	Wu 2010	Wrong patient population;
Profile and severity of the patients of intensive care units: prospective application of the APACHE II index.	DeFreitas 2010	Wrong patient population;
Illness severity scoring for Intensive Care at Middlemore Hospital, New Zealand: past and future.	Mann 2010	Wrong study design;
Developing severity adjusted quality measures for intensive care units.	AlTehewy 2010	Wrong patient population;
An outcome prediction model for adult intensive care.	Umegaki 2010	Wrong patient population;
A simple tool for mortality prediction in burns patients: APACHE III score and FTSA.	Moore 2010	Wrong patient population;
Automating and simplifying the SOFA score in critically ill patients with cancer.	Nates 2010	Wrong patient population;
Validation of four prognostic scores in patients with cancer admitted to Brazilian intensive care units: results from a prospective multicenter study.	Soares 2010	Wrong patient population;
Applicability of the simplified acute physiology score (SAPS 3) in Brazilian hospitals.	SilvaJunior 2010	Wrong setting;
Serial evaluation of SOFA score in a Brazilian teaching hospital.	Anami 2010	Wrong patient population;
APACHE IV versus PPI for predicting community hospital ICU mortality.	Shrope-Mok 2010	Wrong patient population;
The performance of customised APACHE II and SAPS II in predicting mortality of mixed critically ill patients in a Thai medical intensive care unit.	Khwannimit 2009	Wrong patient population;
[APACHE II and ATN-ISS in acute renal failure (ARF) in intensive care unit (ICU) and non-ICU].	Fernandes 2009	Wrong patient population;
External validation of the Simplified Acute Physiology Score (SAPS) 3 in a cohort of 28,357 patients from 147 Italian intensive care units.	Poole 2009	Wrong patient population;
A comparison between the APACHE II and Charlson Index Score for predicting hospital mortality in critically ill patients.	Quach 2009	Wrong patient population;
APACHE III outcome prediction in patients admitted to the intensive care unit after liver transplantation: a retrospective cohort study.	Keegan 2009	Wrong patient population;

Performance of SAPS3, compared with APACHE II and SOFA, to predict hospital mortality in a general ICU in Southern Europe.	Mbongo 2009	Only abstract ;
Subgroup mortality probability models: are they necessary for specialized intensive care units?.	Nathanson 2009	Wrong patient population;
A comparison of SAPS II and SAPS 3 in a Norwegian intensive care unit population.	Strand 2009	Wrong patient population;
Validation of SAPS 3 Admission Score and comparison with SAPS II.	Capuzzo 2009	Wrong patient population;
Mortality probability model III and simplified acute physiology score II: assessing their value in predicting length of stay and comparison to APACHE IV.	Vasilevskis 2009	Wrong patient population;
Prospective validation of the intensive care unit admission Mortality Probability Model (MPM0-III).	Higgins 2009	Wrong patient population;
Performance of six prognostic scores in critically ILL patients receiving renal replacement therapy.	Maccariello 2008	Wrong outcomes;
Comparison of intensive care outcome prediction models based on admission scores with those based on 24-hour data.	Duke 2008	Wrong patient population;
Comparison of the performance of SAPS II, SAPS 3, APACHE II, and their customized prognostic models in a surgical intensive care unit.	Sakr 2008	Wrong patient population;
Serial evaluation of the MODS, SOFA and LOD scores to predict ICU mortality in mixed critically ill patients.	Khwannimit 2008	Wrong patient population;
Sequential organ failure assessment score and comorbidity: valuable prognostic indicators in chronically critically ill patients.	Lee 2008	Wrong patient population;
Validation of the LOD score compared with APACHE II score in prediction of the hospital outcome in critically ill patients.	Khwannimit 2008	Wrong patient population;
Variation in ICU risk-adjusted mortality: impact of methods of assessment and potential confounders.	Kuzniewicz 2008	Wrong outcomes;
[Evaluation of the reproducibility of the data collection for the APACHE II, APACHE III adapted for Spain and the SAPS II in nine intensive care units in Spain].	Dominguez 2008	Wrong patient population;
[Mortality and hospital stay adjusted for severity as indicators of effectiveness and efficiency of attention to intensive care unit patients].	Dominguez 2008	Wrong study design;
Estimation of the mortality risk of surgical intensive care patients based on routine laboratory parameters.	Stachon 2008	Wrong intervention;
[Nursing Activities Score: comparison among the Index APACHE II and the mortality in patients admitted in intensive care unit].	Nogueira 2007	Wrong patient population;
[Prognostic factors for cancer patients in the postanesthetic recovery unit].	Gonzalez-Perez 2007	Wrong patient population;
Evaluation of outcome scoring systems for patients on extracorporeal membrane oxygenation.	Lin 2007	Wrong patient population;
A comparison of three organ dysfunction scores: MODS, SOFA and LOD for predicting ICU mortality in critically ill patients.	Khwannimit 2007	Wrong patient population;
RIFLE classification can predict short-term prognosis in critically ill cirrhotic patients.	Jenq 2007	Wrong patient population;
Predicting the risk of death in patients in intensive care unit.	Saadat-Niaki 2007	Wrong patient population;
ICU discharge APACHE II scores help to predict post-ICU death.	Chen 2007	Wrong outcomes;
A comparison of APACHE II and SAPS II scoring systems in predicting hospital mortality in Thai adult intensive care units.	Khwannimit 2007	Only abstract ;
External validation of prognostic models for critically ill patients required substantial sample sizes.	Peek 2007	Wrong patient population;
Assessing contemporary intensive care unit outcome: an updated Mortality Probability Admission Model (MPM0-III).	Higgins 2007	Wrong patient population;
[Severity of patients admitted to a Brazilian teaching hospital Intensive Care Unit].	Feijo 2006	Wrong patient population;
Prognostic outcomes after the initiation of an electronic telemedicine intensive care unit (eICU) in a rural health system.	Zawada 2006	Only abstract ;
Comparison of severity of illness scoring systems for patients with nosocomial bloodstream infection due to Pseudomonas aeruginosa.	Marra 2006	Wrong patient population;

Performance of standard severity scoring systems for outcome prediction in patients admitted to a respiratory intensive care unit in North India.	Aggarwal 2006	Wrong patient population;
Acute Physiology and Chronic Health Evaluation (APACHE) IV: hospital mortality assessment for today's critically ill patients.	Zimmerman 2006	Wrong patient population;
Prospective cohort study comparing sequential organ failure assessment and acute physiology, age, chronic health evaluation III scoring systems for hospital mortality prediction in critically ill cirrhotic patients.	Chen 2006	Wrong patient population;
A comparison of admission and worst 24-hour Acute Physiology and Chronic Health Evaluation II scores in predicting hospital mortality: a retrospective cohort study.	Ho 2006	Wrong patient population;
SAPS 3--From evaluation of the patient to evaluation of the intensive care unit. Part 2: Development of a prognostic model for hospital mortality at ICU admission.	Moreno 2005	Wrong patient population;
SAPS 3--From evaluation of the patient to evaluation of the intensive care unit. Part 1: Objectives, methods and cohort description.	Metnitz 2005	Wrong outcomes;
Prognostic value of acute physiology and chronic health evaluation II and organ system failure in patients with acute renal failure requiring dialysis.	Wang 2005	Wrong setting;
[Comparing the performance of three severity scoring systems for ICU patients: APACHE III, SAPS II, MPM II].	Kim 2005	Wrong patient population;
Mortality prediction using SAPS II: an update for French intensive care units.	LeGall 2005	Wrong patient population;
Mortality risk factors and validation of severity scoring systems in critically ill patients with acute renal failure.	Lima 2005	Wrong patient population;
External validation of severity scoring systems for acute renal failure using a multinational database.	Uchino 2005	Wrong patient population;
Sequential organ failure predicts mortality of patients with a haematological malignancy needing intensive care.	Cornet 2005	Wrong patient population;
Limited ability of SOFA and MOD scores to discriminate outcome: a prospective evaluation in 1,436 patients.	Zygun 2005	Wrong patient population;
Evaluating the performance of an institution using an intensive care unit benchmark.	Afessa 2005	Wrong patient population;
SAPS II revisited.	Aegerter 2005	Wrong patient population;
Use of the sequential organ failure assessment score as a severity score.	Kajdacsy-BallaAmaral 2005	Only abstract ;
Evaluation of prognostic indexes in critical acute renal failure patients.	Batista 2004	Wrong patient population;
Performance of six severity-of-illness scores in cancer patients requiring admission to the intensive care unit: a prospective observational study.	Soares 2004	Wrong patient population;
Performance evaluation of APACHE II score for an Indian patient with respiratory problems.	Gupta 2004	Wrong patient population;
Prediction of risk of death using 30-day outcome: a practical end point for quality auditing in intensive care.	Graham 2004	Wrong outcomes;
Comparison of the severity of illness scoring systems for critically ill cirrhotic patients with renal failure.	Chen 2004	Wrong patient population;
Comparison of three severity scores for critically ill cancer patients.	Schellongowski 2004	Wrong patient population;
Evaluation of APACHE II system among intensive care patients at a teaching hospital.	Chiavone 2003	Wrong patient population;
Validation of the multiple organ dysfunction (MOD) score in critically ill medical and surgical patients.	Buckley 2003	Wrong patient population;
Simplified Acute Physiology Score III: a project for a new multidimensional tool for evaluating intensive care unit performance.	Vazquez 2003	Wrong study design;
[Intensive care medicine in the Netherlands, 1997-2001. I. Patient population and treatment outcome].	deJonge 2003	Only abstract ;
Intensive care unit support and Acute Physiology and Chronic Health Evaluation III performance in hematopoietic stem cell transplant recipients.	Afessa 2003	Wrong patient population;
Predictive accuracy of severity scoring system: a prospective cohort study using APACHE III in a Korean intensive care unit.	Ihnsook 2003	Wrong study design;
Evaluation of the P-POSSUM mortality prediction algorithm in Australian surgical intensive care unit patients.	Organ 2002	Only abstract ;

The Multiple Organ Dysfunction Score (MODS) versus the Sequential Organ Failure Assessment (SOFA) score in outcome prediction.	PeresBota 2002	Wrong patient population;
Can we predict prognosis using mortality probability model IIo?.	Arabi 2002	Wrong patient population;
Calibration and discrimination by daily Logistic Organ Dysfunction scoring comparatively with daily Sequential Organ Failure Assessment scoring for predicting hospital mortality in critically ill patients.	Timsit 2002	Wrong study design;
Rating the quality of intensive care units: is it a function of the intensive care unit scoring system?.	Glance 2002	Wrong study design;
Integration of APACHE II and III scoring systems in extremely high risk patients with acute renal failure treated by dialysis.	Chen 2002	Wrong patient population;
Comparison of multiple organ dysfunction scores in the prediction of hospital mortality in the critically ill.	Pettila 2002	Wrong patient population;
External validation of a modified model of Acute Physiology and Chronic Health Evaluation (APACHE) II for orthotopic liver transplant patients.	Arabi 2002	Wrong patient population;
["Simplified Acute Physiology Score" (SAPS II) ina the assessment of severity of illness in surgical intensive care patients].	Agha 2002	Only abstract ;
Prospective independent validation of APACHE III models in an Australian tertiary adult intensive care unit.	Cook 2002	Wrong patient population;
Automatic calculation of a modified APACHE II score using a patient data management system (PDMS).	Junger 2002	Wrong outcomes;
Customized prediction models based on APACHE II and SAPS II scores in patients with prolonged length of stay in the ICU.	Suistomaa 2002	Wrong patient population;
[Severity assessment by APACHE III system in Spain].	VazquezMata 2001	Wrong patient population;
Serial evaluation of the SOFA score to predict outcome in critically ill patients.	Ferreira 2001	Wrong patient population;
Predicting mortality in patients suffering from prolonged critical illness: an assessment of four severity-of-illness measures.	Carson 2001	Wrong patient population;
Evaluation of the logistic organ dysfunction system for the assessment of organ dysfunction and mortality in critically ill patients.	Metnitz 2001	Wrong patient population;
Accuracy of a composite score using daily SAPS II and LOD scores for predicting hospital mortality in ICU patients hospitalized for more than 72 h.	Timsit 2001	Wrong study design;
Short-term prognosis in critically ill patients with cirrhosis assessed by prognostic scoring systems.	Wehler 2001	Wrong patient population;
Outcomes and APACHE II predictions for critically ill patients with acute renal failure requiring dialysis.	Chen 2001	Wrong patient population;
Performance of the score systems Acute Physiology and Chronic Health Evaluation II and III at an interdisciplinary intensive care unit, after customization.	Markgraf 2001	Wrong patient population;
Risk stratification in emergency surgical patients: is the APACHE II score a reliable marker of physiological impairment?.	Koperna 2001	Wrong patient population;
Validation of severity scoring systems SAPS II and APACHE II in a single-center population.	Capuzzo 2000	Wrong patient population;
Ratios of observed to expected mortality are affected by differences in case mix and quality of care.	Metnitz 2000	Wrong patient population;
Performance of APACHE III models in an Australian ICU.	Cook 2000	Wrong patient population;
Effect of mortality rate on the performance of the Acute Physiology and Chronic Health Evaluation II: a simulation study.	Glance 2000	Wrong patient population;
Evaluation of the SOFA score: a single-center experience of a medical intensive care unit in 303 consecutive patients with predominantly cardiovascular disorders. Sequential Organ Failure Assessment.	Janssens 2000	Wrong patient population;
[Application of the Simplified Acute Physiology Score II (SAPS II) in a medical intensive care unit].	Ghuysen 2000	Only abstract ;
Assessment of the performance of five intensive care scoring models within a large Scottish database.	Livingston 2000	Wrong patient population;
Predicting patient outcome from acute renal failure comparing three general severity of illness scoring systems.	Fiaccadori 2000	Wrong setting;
An analysis of excess mortality not predicted to occur by APACHE III in an Australian level III intensive care unit.	Buist 2000	Wrong patient population;

Comparison of Acute Physiology and Chronic Health Evaluation II (APACHE II) and Simplified Acute Physiology Score II (SAPS II) scoring systems in a single Greek intensive care unit.	Katsaragakis 2000	Wrong patient population;
Upper gastrointestinal bleeding in patients with hepatic cirrhosis: clinical course and mortality prediction.	Afessa 2000	Wrong patient population;
Comparison of acute physiology and chronic health evaluations II and III and simplified acute physiology score II: a prospective cohort study evaluating these methods to predict outcome in a German interdisciplinary intensive care unit.	Markgraf 2000	Only abstract ;
Use of the logistic organ dysfunction system to study mortality in an Indian intensive care unit.	Sampath 1999	Wrong study design;
Severity and prognosis in intensive care: prospective application of the APACHE II index.	Costa 1999	Wrong patient population;
Development of a new prognostic system and validation of APACHE II for surgical ICU mortality: a multicenter study in Taiwan.	Kuo 1999	Wrong patient population;
Application of mortality prediction systems to individual intensive care units.	Patel 1999	Wrong patient population;
Evaluation of an interdisciplinary data set for national intensive care unit assessment.	Metnitz 1999	Wrong patient population;
Comparison of outcome from intensive care admission after adjustment for case mix by the APACHE III prognostic system.	Pappachan 1999	Wrong study design;
[Prediction of mortality and quality of life in polytraumatized patients: APACHE II versus APACHE III].	CanovasMartinez 1998	Wrong patient population;
[Value of the Hannover Intensive Score (HIS) in internal medicine intensive care].	vonBierbrauer 1998	Wrong patient population;
[Mathematical model for the predictive value of a test in critically ill patients studies according to APACHE II score and pathology at admission].	Donati 1998	Wrong study design;
Predicting outcome in the intensive care unit using scoring systems: is new better? A comparison of SAPS and SAPS II in a cohort of 1,393 patients. GiViTi Investigators (Gruppo Italiano per la Valutazione degli interventi in Terapia Intensiva). Simplified	Bertolini 1998	Wrong patient population;
Evaluation of acute physiology and chronic health evaluation III predictions of hospital mortality in an independent database.	Zimmerman 1998	Wrong patient population;
Predictive value of severity scoring systems: comparison of four models in Tunisian adult intensive care units.	Nouira 1998	Only abstract ;
[Validation of the acute physiology and chronic health evaluation (APACHE) III scoring system and comparison with APACHE II in German intensive care units].	vonBierbrauer 1998	Wrong patient population;
Evaluation of the uniformity of fit of general outcome prediction models.	Moreno 1998	Wrong patient population;
Evaluation of two outcome prediction models on an independent database.	Moreno 1998	Wrong patient population;
[Risk stratification and prognosis in critical surgical patients using the Acute Physiology, Age and Chronic Health III System (APACHE III)].	Carneiro 1997	Wrong patient population;
[Intensity of treatment and severity of illness in the intensive care unit (ICU)].	Capuzzo 1997	Wrong patient population;
Predictors of mortality in a medical intensive care unit.	Eapen 1997	Wrong patient population;
APACHE II in a postoperative intensive care unit in Thailand.	Lertakyamanee 1997	Only abstract ;
Outcome prediction in intensive care: results of a prospective, multicentre, Portuguese study.	Moreno 1997	Wrong patient population;
Predicting mortality in intensive care patients with acute renal failure treated with dialysis.	Douma 1997	Wrong patient population;
Prediction of outcome from intensive care: a prospective cohort study comparing Acute Physiology and Chronic Health Evaluation II and III prognostic systems in a United Kingdom intensive care unit.	Beck 1997	Wrong patient population;
Patient outcome and intensive care resource allocation using APACHE II.	Lim 1996	Only abstract ;
The performance of SAPS II in a cohort of patients admitted to 99 Italian ICUs: results from GiViTI. Gruppo Italiano per la Valutazione degli interventi in Terapia Intensiva.	Apolone 1996	Wrong patient population;
A comparison of the Acute Physiology and Chronic Health Evaluation (APACHE) II score and the Trauma-Injury Severity Score (TRISS) for outcome assessment in intensive care unit trauma patients.	Wong 1996	Wrong patient population;

Application of the APACHE III prognostic system in Brazilian intensive care units: a prospective multicenter study.	Bastos 1996	Wrong patient population;
Mortality predicted by APACHE II. The effect of changes in physiological values and post-ICU hospital mortality.	Goldhill 1996	Wrong outcomes;
The Logistic Organ Dysfunction system. A new way to assess organ dysfunction in the intensive care unit. ICU Scoring Group.	LeGall 1996	Wrong patient population;
Prediction of survival of critically ill patients by admission comorbidity.	Poses 1996	Wrong intervention;
Factors affecting the performance of the models in the Mortality Probability Model II system and strategies of customization: a simulation study.	Zhu 1996	Wrong patient population;
[Severity scores underestimate the seriousness of acute renal failure after emergency surgery].	Frikha 1995	Wrong patient population;
The severity of disease measurements among Thai medical intensive care unit patients.	Kiatboonsri 1995	Only abstract ;
Comparison of APACHE II and III scoring systems for mortality prediction in critical surgical illness.	Barie 1995	Wrong patient population;
[Evaluation of the prognosis of critically ill surgical patients by APACHE II score system].	Wu 1995	Wrong patient population;
The use of APACHE III to evaluate ICU length of stay, resource use, and mortality after coronary artery by-pass surgery.	Becker 1995	Wrong patient population;
[Comparison of APACHE-II AND APACHE-III for classification of disease severity of intensive care patients].	Bein 1995	Only abstract ;
[Assessment of intensive therapy: 4-year experience using the Apache II severity score].	Feri 1995	Wrong patient population;
APACHE II scoring for predicting outcome in cerebral malaria.	Wilairatana 1995	Wrong patient population;
A comparison of severity of illness scoring systems for intensive care unit patients: results of a multicenter, multinational study. The European/North American Severity Study Group.	Castella 1995	Wrong patient population;
Evaluation of predictive ability of APACHE II system and hospital outcome in Canadian intensive care unit patients.	Wong 1995	Wrong patient population;
The predictive value of four scoring systems in liver transplant recipients.	Bein 1995	Wrong patient population;
Use of daily Acute Physiology and Chronic Health Evaluation (APACHE) II scores to predict individual patient survival rate.	Rogers 1994	Only abstract ;
Intensive Care Society's Acute Physiology and Chronic Health Evaluation (APACHE II) study in Britain and Ireland: a prospective, multicenter, cohort study comparing two methods for predicting outcome for adult intensive care patients.	Rowan 1994	Wrong patient population;
Daily prognostic estimates for critically ill adults in intensive care units: results from a prospective, multicenter, inception cohort analysis.	Wagner 1994	Wrong patient population;
Mortality probability models for patients in the intensive care unit for 48 or 72 hours: a prospective, multicenter study.	Lemeshow 1994	Wrong study design;
A prospective comparison of two multiple organ dysfunction/failure scoring systems for prediction of mortality in critical surgical illness.	Barie 1994	Wrong patient population;
Verification of the Acute Physiology and Chronic Health Evaluation scoring system in a Hong Kong intensive care unit.	Oh 1993	Only abstract ;
Validation of APACHE II score in a surgical intensive care unit.	Chen 1993	Wrong patient population;
Acute physiology and chronic health evaluation (APACHE II) scoring in the Medical Intensive Care Unit, National University Hospital, Singapore.	Lee 1993	Wrong patient population;
A new Simplified Acute Physiology Score (SAPS II) based on a European/North American multicenter study.	LeGall 1993	Wrong patient population;
Intensive Care Society's APACHE II study in Britain and Ireland--II: Outcome comparisons of intensive care units after adjustment for case mix by the American APACHE II method.	Rowan 1993	Wrong patient population;
Intensive Care Society's APACHE II study in Britain and Ireland--I: Variations in case mix of adult admissions to general intensive care units and impact on outcome.	Rowan 1993	Wrong patient population;
[Mortality in an intensive care unit: predictive value of APACHE II severity score versus maximum APACHE].	Dougnac 1993	Wrong patient population;
Mortality Probability Models (MPM II) based on an international cohort of intensive care unit patients.	Lemeshow 1993	Wrong patient population;

Evaluation of the consistency of Acute Physiology and Chronic Health Evaluation (APACHE II) scoring in a surgical intensive care unit.	Berger 1992	Only abstract ;
Mortality prediction models in intensive care: acute physiology and chronic health evaluation II and mortality prediction model compared.	Castella 1991	Wrong patient population;
Acute Physiology and Chronic Health Evaluation (APACHE II) score and outcome in the surgical intensive care unit: an analysis of multiple intervention and outcome variables in 1,238 patients.	Rutledge 1991	Wrong patient population;
Critical Care Scoring System--new concept based on hemodynamic data.	Yeung 1990	Only abstract ;
Outcome prediction models on admission in a medical intensive care unit: do they predict individual outcome?.	Schafer 1990	Wrong patient population;
A critical study of the APACHE II scoring system using earlier data collection.	Waters 1990	Wrong patient population;
Systems for scoring severity of illness in intensive care.	Turner 1989	Wrong patient population;
Prediction of outcome from critical illness. A comparison of clinical judgment with a prediction rule.	Brannen 1989	Wrong outcomes;
[Prognostic accuracy and efficacy of treatment at intensive care units evaluated by the APACHE II system].	MilaniJunior 1989	Only abstract ;
[Severity evaluation system: APACHE II, SAPS. National experience in a unit of medical intensive therapy].	Dougnac 1989	Wrong patient population;
Failure of APACHE II alone as a predictor of mortality in patients receiving total parenteral nutrition.	Hopefl 1989	Wrong patient population;
Audit of intensive care: a 30 month experience using the Apache II severity of disease classification system.	Jacobs 1988	Wrong patient population;
Comparison of clinical assessment with APACHE II for predicting mortality risk in patients admitted to a medical intensive care unit.	Kruse 1988	Wrong patient population;
Predicting deaths among intensive care unit patients.	Chang 1988	Wrong patient population;
Validation of the mortality prediction model for ICU patients.	Teres 1987	Wrong patient population;
APACHE-acute physiology and chronic health evaluation: a physiologically based classification system.	Knaus 1981	Wrong outcomes;
Predictive Ability of Scoring Systems for Mortality in Older Adults in Intensive Care Unit of a University Hospital: A Single-Center Retrospective Cohort Study	Azakli 2024	Wrong patient population;
Retrospective Evaluation of the Accuracy of Five Different Severity Scores To Predict the Mortality in Burn Patients	DeCarvalho 2024	Wrong patient population;
The APACHE-II score and the effect of discharge practices on readmission and mortality in intensive care patients	Doganci 2024	Wrong patient population;
Clinical characteristics and mortality prediction of patients admitted to the Hong Kong East Cluster intensive care units in the COVID-19 fifth wave	Man 2024	Wrong patient population;
A tertiary care center-based study of a novel 'ICU Mortality and Prolonged Stay Risk Scoring System'	Widyastuti 2024	Wrong patient population;
External validation of SAPS II score reported to the Norwegian Intensive Care and Pandemic Registry (NIPaR)	Buanes 2023	Wrong patient population;
PROGNOSTIC VALUE OF APACHE II SCORE, SOFA SCORE AND BIOMARKERS IN PATIENTS OF SEPSIS AND SEPTIC SHOCK - A COMPARATIVE STUDY	Rani 2023	Wrong patient population;
Comparison of the Poisoning Severity Score, Sequential Organ Failure Assessment Score, and Acute Physiology and Chronic Health Evaluation II Score with Lactate to assess the outcome in Acute Organophosphorus Poisoning	KrishnaMoorthy 2023	Wrong patient population;
Validation of Sepsis-3 using survival analysis and clinical evaluation of quick SOFA, SIRS, and burn-specific SIRS for sepsis in burn patients with suspected infection	Yoon 2023	Wrong patient population;
Evaluating Prognostic Bias of Critical Illness Severity Scores Based on Age, Gender, and Primary Language in the USA: A Retrospective Multicenter Study	Liu 2022	Wrong outcomes;
Updating mortality risk estimation in intensive care units from high-dimensional electronic health records with incomplete data	Bouvarel 2022	Wrong patient population;
UTILITY OF APACHEII, SAPS II, AND SOFA SCORES AS INDICATORS OF SEVERITY OF SEPSIS AND PREDICTORS OF MORTALITY IN A TERTIARY CARE HOSPITAL	Mittal 2022	Wrong patient population;

Predictive Value of Sequential Organ Failure Assessment (SOFA), Quick Sequential Organ Failure Assessment (qSOFA), Acute Physiology and Chronic Health Evaluation (APACHE II), and New Early Warning Signs (NEWS-2) Scores Estimate Mortality of COVID-19 Patie	Asmarawati 2022	Wrong patient population;
SOFAMONIA: Comparison of the original SOFA score with the proposed new score including serum ammonia	Zanuto 2021	Wrong outcomes;
Categorical Apache IV prediction of ICU and 90 day mortality	Bergmans 2021	Wrong patient population;
Comparison of Sensitivity, Specificity and Accuracy of APACHE II, SAPS II and SOFA Scoring Systems as Predictors of Mortality in ICU Patients	Furqan 2021	Wrong patient population;
NUTRIC-S proposal: Using SAPS 3 for mortality prediction in nutritional risk ICU patients	Toledo 2020	Wrong patient population;
Erratum: The use of APACHE II, SOFA, SAPS 3, C-reactive protein/albumin ratio, and lactate to predict mortality of surgical critically ill patients: A retrospective cohort study: Erratum (Medicine (2019) 98 26 (e16204))	Anonymous 2019	Wrong patient population;
Comparison of APACHE II and SAPS II scoring systems in prediction of critically ill patient's outcome	Aminiahidashti 2019	Wrong patient population;
Combined anatomic and physiologic scoring systems for predicting in-hospital mortality in ICU patients with severe trauma: A multicenter observational cohort study	Ma 2019	Wrong patient population;
Performance of the Acute Physiology and Chronic Health Evaluation II (APACHE II) in the prediction of hospital mortality in a mixed ICU in Singapore	Lew 2019	Wrong study design;
Application of APACHE-II and SOFA score as a predictive outcome in Ramathibodi surgical intensive care unit	Pornwaragron 2019	Wrong outcomes;
Comparison of mortality estimation by using the disease severity standardized scoring systems of APACHE II GSC and APACHE II 4 score	Sejahrood 2018	Wrong patient population;
Accuracy and performance assessment of apache iv and saps 3 in geriatric patients admitted to the intensive care unit	KorkmazToker 2018	Wrong patient population;
Clinical effectiveness of modified sequential organ failure assessment scoring system for predicting ICU indexing scores	Babamohamadi 2016	Wrong patient population;
Single-centre validation of the EASL-CLIF Consortium definition of acute-on-chronic liver failure and CLIF-SOFA for prediction of mortality in cirrhosis	Silva 2015	Wrong patient population;
Evaluation of probability of survival using APACHE II and TRISS method in orthopaedic polytrauma patients in a tertiary care centre	Agarwal 2015	Wrong patient population;
Sequential Organ Failure Assessment (SOFA) score as a predictor of outcome in patients admitted in a medical ICU	Faraz 2015	Wrong study design;
Validation of SAPS-3 and APACHE-III in Mediterranean area	Rivera-Lopez 2014	Wrong patient population;
Prognostic usefulness of sabadell score in critically ill patients hospitalized at internal medicine service	Nunez-Armendariz 2014	Wrong patient population;
Comparing acute physiology and chronic health evaluation (APACHE) IV and simplified acute physiology (SAPA) III scoring methods in predicting mortality rate in patients admitted to intensive care unit	Yaghoubi 2014	Wrong patient population;
Evaluation of patient mortality in intensive care units using the APACHE IIscoring system	Soleimani 2014	Wrong patient population;
Why the surgical patients are so critical in their intensive care unit arrival?	Basile-Filho 2013	Wrong patient population;
Clinical accuracy of RIFLE and acute kidney injury network (AKIN) criteria for predicting hospital mortality in critically Ill patients with multi-organ dysfunction syndrome	Ratanarat 2013	Wrong intervention;
Verification of validity of mpm ii for neurological patients in intensive care units	Kim 2011	Wrong patient population;
Validation of six mortality prediction systems for ICU surgical populations	Timmers 2011	Only abstract ;
The effectiveness of scoring systems and various biochemical parameters in predicting survival in a respiratory intensive care unit	Yildiz 2010	Wrong patient population;
Profile and severity of the patients of intensive care units: Prospective application of the APACHE II index	deFreitas 2010	Wrong patient population;
Comparison of Apache II, SOFA, and modified SOFA scores in predicting mortality of surgical patients in intensive care unit at Dr. Hasan Sadikin General Hospital	Halim 2009	Wrong patient population;

The predictive capability of APACHE II score in determining mortality among critically ill surgical population	Hashem 2008	Wrong study design;
Combining Sequential Organ Failure Assessment (SOFA) score with Acute Physiology and Chronic Health Evaluation (APACHE) II score to predict hospital mortality of critically ill patients	Ho 2007	Wrong patient population;
Validating the use of the APACHE II score in a tertiary South African ICU	vanderMerwe 2005	Wrong patient population;
Modified organ system failure score for critically III patients with acute renal failure requiring dialysis	Chen 2003	Wrong patient population;
Can we measure ICU performance with the SAPS II?	Aegerter 2003	No English version available;
Customised prediction models based on APACHE II and SAPS II scores in patients with prolonged length of stay in the ICU	Suistomaa 2002	Wrong patient population;
APACHE III scoring system in critically III patients with acute renal failure requiring dialysis	Chen 2002	Wrong patient population;
Use of the simplified acute physiology score II (SAPS II) in a medical intensive care unit	Ghuysen 2000	Wrong patient population;
Validation of Mortality Probability Models II (MPM II) at admission (MPM II-0), at 24 hours (MPM 11-24), and at 48 hours (MPM II-48) compared with the hospital mortality predictions from APACHE II and SAPS II measured in the first and second days of ICU s	SerranoHernandez 2000	Wrong patient population;
The use of maximum SOFA score to quantify organ dysfunction/failure in intensive care. Results of a prospective, multicentre study	Moreno 1999	Wrong patient population;
Hannover Intensive Score (HIS) in medical care medicine	VonBierbrauer 1998	Only abstract ;
Use of the SOFA score to assess the incidence of organ dysfunction/failure in intensive care units: Results of a multicenter, prospective study	Vincent 1998	Wrong patient population;
Scoring systems. Validation of APACHE III and comparison to APACHE II in a German intensive care unit	VonBierbrauer 1998	Wrong patient population;
Severity scores underestimate the seriousness of acute renal failure following emergency surgery	Frikha 1995	Wrong patient population;
Comparison of two severity-of-disease classification systems (APACHE II and APACHE III) in critically ill patients	Bein 1995	Wrong patient population;
A comparison of severity of illness scoring systems for intensive care unit patients: Results of a multicenter, multinational study	Castella 1995	Only abstract ;
Scope and limitations of score systems in intensive-care medicine	Bein 1993	Wrong outcomes;
A simplified severity index for intensive care patients. Results of a prospective study in 280 cases	Bedock 1985	Only abstract ;
Adverse Sequential Organ Failure Assessment Score as a Predictor of Mortality in Patients Requiring Critical Care in Pakistan	Janjua 2024	Wrong patient population;
The Sequential Organ Failure Assessment (SOFA) Score: has the time come for an update?	Moreno 2023	Wrong outcomes;
Construction and evaluation of a risk prediction model for pulmonary infection-associated acute kidney injury in intensive care units	Cao 2023	Wrong outcomes;
Comparison of 6 Mortality Risk Scores for Prediction of 1-Year Mortality Risk in Older Adults with Multimorbidity	Schneider 2022	Wrong patient population;
Serial Evaluation of Sequential Organ Failure Assessment Score in Predicting 1-Year Mortality in Critically Ill Patients	Ralib 2022	Wrong patient population;
An easy-to-use nomogram for predicting in-hospital mortality risk in COVID-19: a retrospective cohort study in a university hospital	Acar 2021	Wrong patient population;
Regional performance variation in external validation of four prediction models for severity of COVID-19 at hospital admission: An observational multi-centre cohort study	Wickstr√∏m 2021	Wrong patient population;
Validation of prognostic scores in extracorporeal life support: A multi-centric retrospective study	Fisser 2021	Wrong patient population;
Are prognostic tools losing accuracy? Development and performance of a novel age-calibrated severity scoring system for critically ill patients	Menezes 2020	Wrong study design;
Validation of END-of-life ScorING-system to identify the dying patient: A prospective analysis	Villa 2020	Wrong intervention;

The SOFA score - Development, utility and challenges of accurate assessment in clinical trials	Lambden 2019	Wrong outcomes;
Mortality of patients with acute kidney injury requiring renal replacement therapy	Czempik 2018	Wrong patient population;
Predicting mortality and hospitalization of older adults by the multimorbidity frailty index	Wen 2017	Wrong intervention;
Using patient admission characteristics alone to predict mortality of critically ill patients: A comparison of 3 prognostic scores	Ho 2016	Wrong patient population;
Evaluation of Acute Physiology and Chronic Health Evaluation II and sequential organ failure assessment scoring systems for prognostication of outcomes among Intensive Care Unit,Ä≤s patients	Hosseini 2016	Wrong patient population;
Scoring systems in assessing survival of critically ill ICU patients	Sekulic 2015	Wrong patient population;
External validation of the intensive care national audit & research centre (ICNARC) risk prediction model in critical care units in Scotland	Harrison 2014	Wrong patient population;
Development and validation of the critical care outcome prediction equation, version 4	Duke 2013	Wrong patient population;
The use of infection probability score and sequential organ failure assessment scoring systems in predicting mechanical ventilation requirement and duration	Honarmand 2009	Wrong outcomes;
Severity of illness and outcome in ICU patients in the Netherlands: Results from the NICE registry 2006-2007	deLange 2009	Wrong patient population;
SAPS 3 admission score: An external validation in a general intensive care population	Ledoux 2008	Wrong patient population;
Predicting death and readmission after intensive care discharge	Campbell 2008	Wrong setting;
Apache II and Apache III prognostic markers. Experience in three Mexican intensive care units	Olivares-Dur√°n 2005	Only abstract ;
A comparison of admission and worst 24-hour Acute Physiology and Chronic Health Evaluation II scores in predicting hospital mortality: A retrospective cohort study	Ho 2005	Wrong study design;
Reliability and accuracy of Sequential Organ Failure Assessment (SOFA) scoring	Arts 2005	Wrong outcomes;
Multiple organ system failure in critically ill cirrhotic patients: A comparison of two multiple organ dysfunction/failure scoring systems	Tsai 2004	Wrong patient population;
Organ system failure scoring system can predict hospital mortality in critically III cirrhotic patients	Tsai 2003	Wrong patient population;
Role of serum creatinine and prognostic scoring systems in assessing hospital mortality in critically ill cirrhotic patients with upper gastrointestinal bleeding	Chen 2003	Wrong patient population;
The SOFA score to evaluate organ failure and prognosis in the intensive care unit patients	Kim 2004	Wrong patient population;
Validation of organ failure scoring systems in objective illness characterization	Siemiatkowski 2002	Wrong outcomes;
Assessment of performance of four mortality prediction systems in a Saudi Arabian intensive care unit	Arabi 2002	Wrong patient population;
Use of the Simplified Acute Physiology Score (SAPS II) for assessment of disease severity in surgical intensive care patients	Agha 2002	Wrong outcomes;
Unidad de terapia intensiva en pos de mejorar calidad de vida y no de prolongar agon√≠a	Gambino 2000	Wrong patient population;
Customization of SAPS II for the assessment of severity in Italian ICU patients. ARCHIDIA. Archivio Diagnostico.	Sicignano 2000	Only abstract ;
Comparison of different scoring systems (APACHE III, SAPSII, MPM II0-72): Value of daily measurement in 303 consecutive patients	Janssens 1999	Wrong patient population;
Intensive care improves patient survival	Sprung 1999	Wrong patient population;
Statistical modeling of prognostic indices	Livianu 1999	Wrong study design;
Comparison of SAPS II, MPM II24 and SAPS in intensive care	Cominotti 1999	Wrong patient population;
How changes in SOFA score can predict out-come	Ferreira 1999	Wrong outcomes;

Predictive value of severity scoring systems: comparison of four models in three mexican intensive care units included in the multicenter database of intensive care	Ceron 1999	Wrong study design;
Prognostic scoring for critically ill hospitalized patients.	Ahluwalia 1999	Wrong patient population;
Comparison of APACHE II and day 1 multiple organ dysfunction score in critically ill medical patients	Matchett 1999	Wrong patient population;
Comparison of 3 severity of illness scoring ststems for imtemsivk cari unit (icu) patients	Livxanu 1998	Only abstract ;
Comparison of severity systems (APACHE II, SAPS II, and MPM II) in ICU patients	Lin 1998	Wrong patient population;
An IC;D-9 based illness severity score (ICISS) out performs apache II in predicting survival, hospital charges and length of stay in surgical intensive care unit patients	Huynh 1998	Wrong patient population;
Prognostic value of first-day and seventh-day score of APACHE III scoring system in critically ill medical patients	Yang 1998	Wrong patient population;
The correlation of organ failure indices with in-hospital mortality	Afessa 1998	Wrong study design;
Predicting Outcome in the Intensive Care Unit Using Scoring Systems: Is New Better? A Comparison of SAPS and SAPS II in a Cohort of 1,393 Patients	Bertolini 1998	Wrong patient population;
Predictive value of a combined physiologic-therapeutic scoring system (MARIS) and comparison to only physiologically (APACHE II) or the therapeutically (TISS) based systems in medical intensive care medicine	VonBierbrauer 1997	Wrong patient population;
Evaluation of different score systems for the prognosis of morbidity and mortality on an anaesthesiological ICU	Schulze 1996	Wrong patient population;
The Influence of length of stay in the ICU on power of discrimination of a multipurpose severity score (SAPS)	Sicignano 1996	Wrong outcomes;
Evaluation of severity scoring systems in ICUs-translation, conversion and definition ambiguities as a source of inter-observer variability in Apache II, SAPS and OSF	F√©ry-Lemonnier 1995	Wrong patient population;
Predicting outcome in ICU patients	Suter 1994	Wrong patient population;
Use of APACHE II classification to evaluate outcome and response to therapy in acute renal failure patients in a surgical intensive care unit	vanBommel 1995	Wrong patient population;
Prospective comparison of clinical judgment and apache ii score in predicting the outcome in critically 111 surgical patients	Meyer 1992	Wrong patient population;
The APACHE III prognostic system: Risk prediction of hospital mortality for critically III hospitalized adults	Knaus 1991	Wrong patient population;
Determinants of immediate survival among chronic respiratory insufficiency patients admitted to an intensive care unit for acute respiratory failure; A prospective multicenter study	Portier 1992	Wrong patient population;
A comparison of APACHE II and a clinical sickness score: A study of 97 consecutive admissions to a District General Hospital Intensive Care Unit	SINCLAIR 1991	Wrong patient population;
Use of the APACHE II scoring method for registering patients receiving intensive care at a central hospital	Hartmann-Andersen 1989	Wrong outcomes;
A comparison of methods to predict mortality of intensive care unit patients	Lemeshow 1987	Wrong patient population;
APACHE II: A severity of disease classification system	Knaus 1985	Wrong patient population;
One year's experience with the APACHE II severity of disease classification system in a general intensive care unit	JACOBS 1987	Wrong patient population;

Table S5. Characteristics of selected ICU severity of illness scoring systems and kidney-related parameters.

Scoring Systems	Year Published	Kidney aspects	Time of score calculation	Variables
**APACHE II**	1985	Creatinine, Chronic dialysis	Worst value from preceding 24 hours since ICU admission	Age<br>Clinical variables:<br>Non-operative or emergency post-op<br>Elective post-op<br>Cirrhosis with portal hypertension, encephalopathy, or hepatic failure<br>NYHA Class IV angina<br>Chronic hypoxia, or chronic restrictive/obstructive lung disease, or respiratory dependency<br>Increased CO_2 or Polycythemia<br>Chronic dialysis<br>Immunocompromised (e.g., immune-suppression, chemotherapy, radiation)<br>Temperature<br>Mean arterial pressure<br>Heart Rate<br>Respiratory rate<br>Oxygenation<br>Arterial pH<br>Serum sodium, potassium,<br>Creatinine<br>Hematocrit<br>White blood cells<br>Glascow Coma Scale
**APACHE III**	1991	Creatinine, BUN, urine output	First 24 hours post ICU admission	Age<br>Comorbid factors:<br>AIDS<br>Hepatic failure<br>Lymphoma<br>Metastatic solid tumour<br>Leukemia/multiple myeloma<br>Immunosuppression<br>Cirrhosis

				ICU admission diagnosis<br>Medical<br>Elective surgical<br>Emergency surgical<br>Most abnormal value within initial 24 hours is used to calculate score:<br>Heat rate<br>Mean Arterial Pressure<br>Temperature<br>Respiratory rate<br>$PaO_2/P(A-a)O_2$<br>Hematocrit<br>White blood cells<br>Sodium, potassium, creatinine, BUN, albumin, bilirubin<br>Urine output<br>Glucose<br>Arterial pH<br>Glascow Coma Scale
**SAPS II**	1994	serum urea or BUN,<br>urine output	First 24 hours post ICU admission	Age<br>Type of admission<br>Scheduled surgical<br>Unscheduled surgical<br>Medical<br>Comorbidities:<br>AIDS<br>Hematologic malignancy<br>Metastatic cancer<br>Heart rate<br>Systolic blood pressure<br>Temperature<br>PaO_2/FiO_2 (if ventilated)<br>Urine output<br>Serum urea or BUN, potassium, sodium, bicarbonate, bilirubin<br>White blood cells<br>Glascow Coma Scale

**SAPS III**	2005	Creatinine	Within 1 hour of ICU admission	Age<br>Length of stay before ICU admission (days)<br>Intrahospital location before ICU admission:<br>Emergency room<br>Other ICU<br>Ward<br>Use of major therapeutic options before ICU admission:<br>Vasoactive drugs<br>Other/none<br>Planned/unplanned ICU admission<br>Surgical status at ICU admission<br>Scheduled surgery<br>No surgery<br>Unplanned surgery<br>Comorbid factors:<br>Cancer therapy<br>NYHA class IV heart failure<br>Hematological cancer<br>Cirrhosis<br>AIDS<br>Metastatic cancer<br>Reason for ICU admission:<br>Cardiovascular<br>Neurological<br>Renal<br>Respiratory<br>Hepatic<br>Haematological<br>Metabolic<br>Digestive<br>Severe trauma<br>Other<br>Oxygenation (PaO_2 , FiO_2)<br>Temperature<br>Heart rate<br>Systolic blood pressure<br>Creatinine

				Total bilirubin<br>Arterial pH<br>Leukocytes, platelets<br>Glascow Coma Scale
**SOFA**	1996	creatinine or urine output	Every 24 hours post ICU admission	Respiratory:<br>PaO_2/FiO_2<br>Respiratory support<br>Coagulation:<br>Platelets<br>Liver:<br>Bilirubin<br>Cardiovascular:<br>Mean arterial pressure<br>Vasopressors required (dopamine, dobutamine, norepinephrine/epinephrine)<br>Central Nervous System:<br>Glascow Coma Scale<br>Renal:<br>Creatinine or Urine output

Abbreviations: AIDS = acquired immunodeficiency syndrome, APACHE = Acute Physiology and Chronic Health Evaluation, BUN = Blood Urea Nitrogen, CO_2 = Carbon dioxide, FiO_2 = Fraction of inspired oxygen, ICU = Intensive Care Unit, NYHA = New York Heart Association, $P(A\text{-}a)O_2$ = Alveolar-arterial oxygen gradient, PaO_2 = Partial pressure of oxygen in arterial blood, pH = hydrogen ion concentration, SAPS = Simplified Acute Physiology Score, SOFA = Sequential Organ Failure Assessment

Table S6. Statistical results summary of outcomes and GRADE assessment.

Outcomes	Number of studies	Overall Risk of Bias	Quality of Evidence (GRADE)
**Discriminatory Accuracy for ESKD Patients**			
APACHE II	4	High	**Low**
APACHE III	2	High	**Low**
SAPS II	3	High	**Low**
SOFA	3	High	**Very low**
**Discriminatory Accuracy for Kidney Transplant Patients**			
SAPS III	2	High	**Very low**
SOFA	2	High	**Very low**

Abbreviations: APACHE = Acute Physiology and Chronic Health Evaluation; GRADE = Grading of Recommendations Assessment, Development and Evaluation; SAPS = Simplified Acute Physiology Score, SOFA = Sequential Organ Failure Assessment

GRADE Judging the certainty of discrimination performance estimates of prognostic models

High quality: Further research is very unlikely to change our confidence in the estimate of effect.
Moderate quality: Further research is likely to have an important impact on our confidence in the estimate of effect and may change the estimate.
Low quality: Further research is very likely to have an important impact on our confidence in the estimate of effect and is likely to change the estimate.
Very low quality: We are very uncertain about the estimate.

Table S7. JBI critical appraisal results for cohort studies.

Study (year)	Q1	Q2	Q3	Q4	Q5	Q6	Q7	Q8	Q9	Q10	Q11	Total* (%)
**Rana et al. (2024)**	N/A	N/A	0.5	1	1	1	0.5	0.5	0.5	N/A	0	5/8 (62.5 %)
**Shimada et al. (2024)**	1	1	1	0.5	0.5	1	1	1	1	N/A	1	9/10 (90.0 %)
**Zhang et al. (2022)**	N/A	N/A	1	1	1	1	1	1	0.5	0	1	7.5/9 (83.3 %)
**Freitas et al. (2018)**	N/A	1	1	1	1	1	1	0.5	0.5	0	1	8/10 (80.0 %)
**Goswami et al. (2018)**	N/A	N/A	0	0.5	0.5	1	1	1	0.5	N/A	1	5.5/8 (68.7. %)
**Akbas et al. (2015)**	0.5	1	0.5	0.5	0.5	1	1	1	1	N/A	1	8/10 (80.0 %)
**Oliveira et al. (2013)**	0.5	1	1	0.5	0.5	1	1	1	1	N/A	1	8.5/10 (85.0 %)
**Juneja et al. (2010)**	N/A	N/A	1	0.5	0	1	1	1	1	N/A	0.5	6/8 (75.0 %)
**Manhes et al. (2005)**	N/A	N/A	0.5	1	1	1	1	1	0.5	0	1	7/9 (77.8 %)
**Dara et al. (2004)**	N/A	N/A	0.5	0.5	0	1	1	1	1	N/A	0.5	5.5/8 (68.8 %)
**Uchino et al. (2003)**	1	1	1	0.5	0.5	1	1	1	0	0	1	8/10 (80.0 %)
**Clermont et al. (2002)**	1	1	1	0.5	0	1	1	1	1	N/A	0.5	8/10 (80.0 %)

1 = Yes, 0= No, 0.5 = Unclear, N/A = not applicable * Total score is calculated after removing any inapplicable items

JBI critical appraisal checklist for cohort studies

Q1 Were the groups similar and recruited from the same population?
Q2 Were the exposures measured similarly to assign people to both exposed and unexposed groups?
Q3 Was the exposed measured in a valid and reliable way?
Q4 Were confounding factors identified?
Q5 Were strategies to deal with confounding factors stated?
Q6 Were the groups/participants free of the outcomes at the start of the study (or at the moment of exposure)?
Q7 Were the outcomes measured in a valid and reliable way?
Q8 Was the follow-up time reported and sufficient to be long enough for outcomes to occur?
Q9 Was follow-up complete, and if not, were the reasons to loss to follow-up described and explored?
Q10 Were strategies to address incomplete follow-up utilized?
Q11 Was appropriate statistical analysis used?

Table S8. PROBAST risk of bias results

Study (year)	Participants	Predictors	Outcome	Analysis	Overall ROB	Applicability concerns
**Rana et al. (2024)**	+	+	?	_	_	_**
**Shimada et al. (2024)**	+	+	+	+	+	+
**Zhang et al. (2022)**	+	_	+	_	_	+
**Freitas et al. (2018)**	+	+	+	_	_	_**
**Goswami et al. (2018)**	+	+	+	_	_	_**
**Akbas et al. (2015)**	+	+	+	_	_	_**
**Oliveira et al. (2013)**	+	+	+	_	_	+
**Juneja et al. (2010)**	+	+	+	_	_	+
**Manhes et al. (2005)**	+	+	+	_	_	+
**Dara et al. (2004)**	+	+	+	_	_	+
**Uchino et al. (2003)**	+	+	+	_	_	+
**Clermont et al. (2002)**	+	+	+	_	_	+

Abbreviations: PROBAST = Prediction model Risk Of Bias Assessment Tool; ROB = risk of bias.

* + indicates low ROB/low concern regarding applicability.
− indicates high ROB/high concern regarding applicability.
? indicates unclear ROB/unclear concern regarding applicability.

** Unclear or inappropriate age specification (i.e., inclusion of patients <18 years old or lack of reporting of age criteria)

Figure S1. Risk of Bias and applicability assessment of included studies using the PROBAST tool.

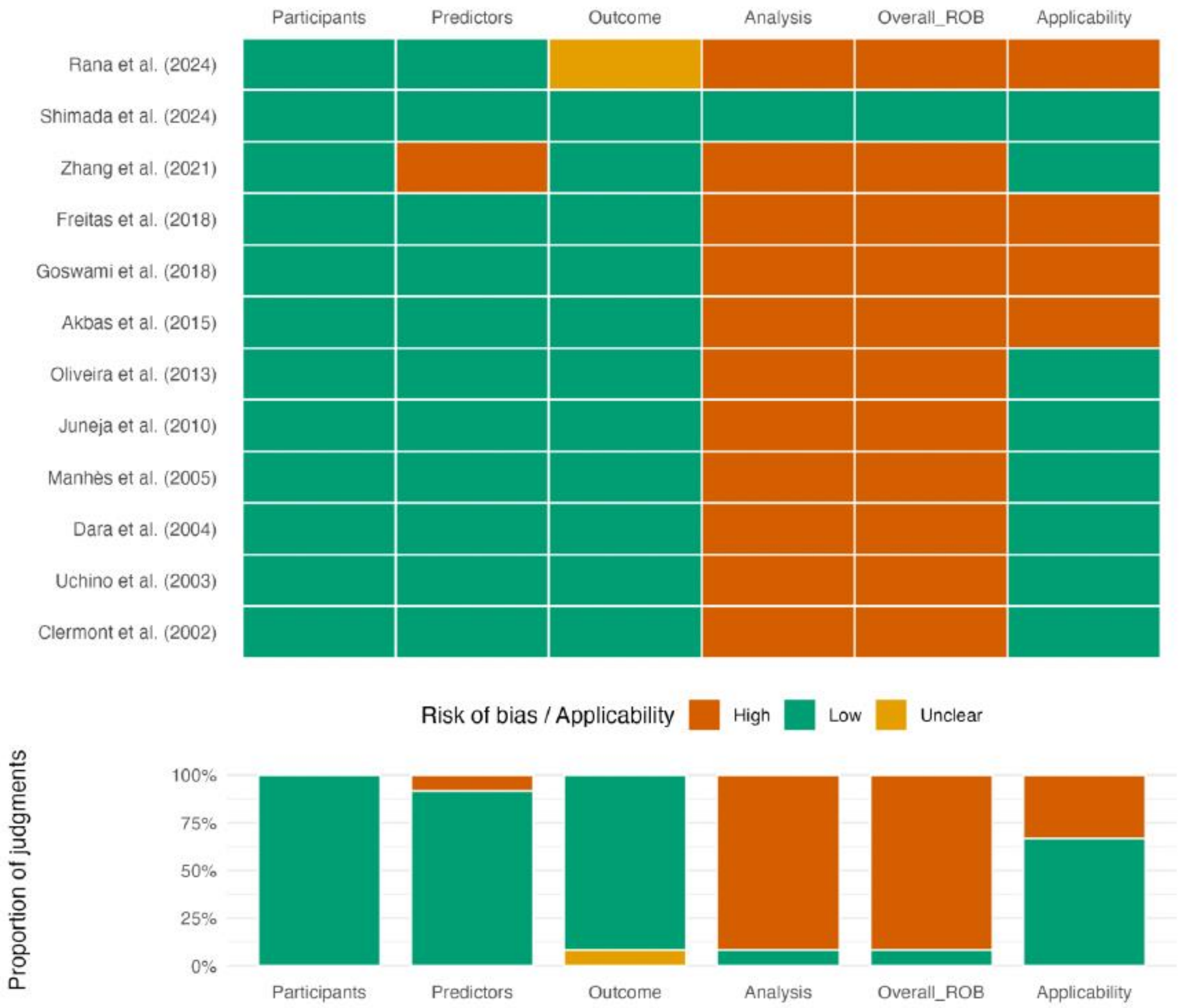

The top panel provides a summary of each domain. The bottom bar plot displays the overall assessment of studies rated as low, unclear, or high risk of bias or applicability concerns.

Abbreviations: PROBAST, Prediction model Risk Of Bias ASsessment Tool; ROB, risk of bias.
